# Comparing MRI metrics to quantify white matter microstructural damage in multiple sclerosis

**DOI:** 10.1002/hbm.24568

**Published:** 2019-03-19

**Authors:** Ilona Lipp, Derek K. Jones, Sonya Bells, Eleonora Sgarlata, Catherine Foster, Rachael Stickland, Alison E. Davidson, Emma C. Tallantyre, Neil P. Robertson, Richard G. Wise, Valentina Tomassini

**Affiliations:** ^1^ Division of Psychological Medicine and Clinical Neurosciences Cardiff University School of Medicine Cardiff UK; ^2^ Cardiff University Brain Research Imaging Centre School of Psychology Cardiff UK; ^3^ Department of Neurophysics Max Planck Institute for Human Cognitive and Brain Sciences Leipzig Germany; ^4^ Mary MacKillop Institute for Health Research Australian Catholic University Melbourne Australia; ^5^ Neurosciences and Mental Health The Hospital for Sick Children Toronto Canada; ^6^ Department of Human Neurosciences Sapienza University of Rome Rome Italy; ^7^ Helen Durham Centre for Neuroinflammation University Hospital of Wales Cardiff UK

**Keywords:** brain MRI, diffusion, lesions, magnetisation transfer ratio, multiple sclerosis, myelin water fraction

## Abstract

Quantifying white matter damage in vivo is becoming increasingly important for investigating the effects of neuroprotective and repair strategies in multiple sclerosis (MS). While various approaches are available, the relationship between MRI‐based metrics of white matter microstructure in the disease, that is, to what extent the metrics provide complementary versus redundant information, remains largely unexplored. We obtained four microstructural metrics from 123 MS patients: fractional anisotropy (FA), radial diffusivity (RD), myelin water fraction (MWF), and magnetisation transfer ratio (MTR). Coregistration of maps of these four indices allowed quantification of microstructural damage through voxel‐wise damage scores relative to healthy tissue, as assessed in a group of 27 controls. We considered three white matter tissue‐states, which were expected to vary in microstructural damage: normal appearing white matter (*NAWM*), T2‐weighted hyperintense lesional tissue without T1‐weighted hypointensity (*T2L*), and T1‐weighted hypointense lesional tissue with corresponding T2‐weighted hyperintensity (*T1L*). All MRI indices suggested significant damage in all three tissue‐states, the greatest damage being in *T1L*. The correlations between indices ranged from *r* = 0.18 to *r* = 0.87. MWF was most sensitive when differentiating *T2L* from *NAWM*, while MTR was most sensitive when differentiating *T1L* from *NAWM* and from *T2L*. Combining the four metrics into one, through a principal component analysis, did not yield a measure more sensitive to damage than any single measure. Our findings suggest that the metrics are (at least partially) correlated with each other, but sensitive to the different aspects of pathology. Leveraging these differences could be beneficial in clinical trials testing the effects of therapeutic interventions.

## 1 INTRODUCTION

Accurate quantification of white matter damage in multiple sclerosis (MS) is important for the development of reparative and neuroprotective strategies (Barkhof, Calabresi, Miller, & Reingold, 2009). While the hallmark of white matter pathology in MS is focal demyelinating lesions, the correlation between the volume of lesional tissue and disability is low, representing an example of the so‐called clinical‐radiological paradox (Barkhof, 1999, 2002). Instead, the severity of damage within lesions and the diffuse microstructural damage outside lesions are factors that appear to better explain disability and prognosis (Filippi, 2001; Giorgio et al., 2010; Kitzler et al., 2012; S. Kolind et al., 2012; Moll et al., 2011).

In MS, white matter microstructure can be compromised by both demyelination and axonal loss. While axonal loss can result directly from inflammation within lesions, it can also occur as a consequence of demyelination outside lesions (Bitsch, Schuchardt, Bunkowski, Kuhlmann, & Bru, 2000; Trapp et al., 1998; Trapp & Stys, 2009). Therefore, the promotion of repair through remyelination and neuroprotection represents the two major goals of novel therapeutic interventions in MS. To improve the characterisation of damage and quantification of repair, non‐invasive MRI‐based methods to accurately measure microstructural damage are crucial. A variety of MRI‐based approaches that probe various physical properties of the tissue have been shown to be sensitive to demyelination and axonal loss in MS (Filippi, Preziosa, & Rocca, 2017; Mallik, Samson, Wheeler‐Kingshott, & Miller, 2014).

One frequently used method to probe white matter microstructure is diffusion tensor imaging (DTI, Basser, Mattiello, and LeBihan (1994)). Membranes and myelin sheaths of the axons collectively hinder diffusion, leading to anisotropic diffusion which, in DTI, is represented by a tensor. Parameters extracted from DTI, such as fractional anisotropy (FA), radial diffusivity (RD), and axial diffusivity (AD), correlate with histological measures of myelination as well as with axonal density (Chang et al., 2017; Klawiter et al., 2011; Moll et al., 2011; Mottershead et al., 2003; Schmierer et al., 2007, 2008). Even though these metrics are sensitive to changes in myelination, diffusion MRI itself is an indirect approach to measure demyelination. Water trapped in the myelin sheaths has a very short T2 relaxation time (Mackay et al., 1994) and does not contribute to the signal measured in diffusion‐weighted sequences because of their comparatively long echo time. Also, myelin is not a prerequisite for diffusion anisotropy (Beaulieu, 2002).

More direct approaches to assessing myelination are myelin water imaging and magnetisation transfer (MT) imaging. Myelin water imaging exploits the signal derived from water that is trapped within myelin sheaths and that has a short T2 relaxation time (between 10 and 55 ms) compared to water in other tissue compartments (Mackay et al., 1994). By making use of a multi‐echo‐ (Mackay et al., 1994) or multi‐flip‐angle strategy (Deoni, Rutt, & Jones, 2008), the relative contribution to the signal from this T2 species can be calculated and is referred to as myelin water fraction (MWF). By contrast, MT imaging makes use of the MT that happens between macromolecules, such as those found in myelin, and surrounding tissue water, when the macromolecular protons are subjected to an off‐resonance radio‐frequency (RF) pulse that selectively saturates the macromolecular pool of protons. Both MWF (Laule et al., 2008; Laule, Leung, Traboulsee, Paty, & Mackay, 2006; Schmierer, Scaravilli, Altmann, Barker, & Miller, 2004) and magnetisation transfer ratio (MTR) (Gareau, Rutt, Karlik, & Mitchell, 2000; Moll et al., 2011; Mottershead et al., 2003; Schmierer et al., 2008) correlate with the histological markers of myelin.

Although these MRI methods are considered valid approaches for assessing microstructural integrity, the relationship between their metrics, that is, to what extent the metrics provide complementary versus redundant information, remains largely unexplored. In healthy volunteers, significant correlations between diffusion‐based metrics and MWF have been reported (De Santis, Drakesmith, Bells, Assaf, & Jones, 2014), but these depend on the brain region investigated and the health of the tissue (Bells, Morris, & Vidarsson, 2007; Mädler, Drabycz, Kolind, Whittall, & Mackay, 2008). Kolind et al. (2008) found low correlations between diffusion‐based metrics and MWF in MS lesions. Also, correlations between MWF and MT‐derived metrics are low or inconsistent between various types of tissue (Underhill, Yuan, & Yarnykh, 2009; Vavasour, Laule, Li, Traboulsee, & MacKay, 2011).

One challenge when comparing DTI‐based metrics with other metrics is that the diffusion tensor is heavily influenced by the underlying fibre architecture (De Santis et al., 2014; Pierpaoli, Chiro, Basser, & Trace, 1996). For example, in two voxels with identical axonal density and myelin content, DTI‐derived metrics may disagree, if one voxel lies in a region with one predominant fibre orientation, while the other voxel lies in a region containing several crossing fibre populations. In this study, we considered the potential spatial heterogeneity of the relationship between white matter MRI metrics and pathology. We z‐transformed the microstructural parameters derived from MS patients to the average parameter of healthy controls in the same location. The resulting voxel‐wise damage scores represent the scaled versions of the original microstructural parameters, which are independent of the underlying fibre architecture. We aimed to explore the relationship between four microstructural metrics (FA, RD, MTR, and MWF) that are frequently used in clinical studies of MS patients. Microstructural damage estimates from the four metrics were considered for three MS tissue‐states: **(a)** normal appearing white matter (*NAWM*), with the least expected damage; **(b)** lesional tissue that only appears hyperintense on a T2‐weighted scan; and **(c)** lesional tissue that also shows T1 hypointensity and is expected to have the strongest underlying microstructural damage (Moll et al., 2011; Sahraian, Radue, Haller, & Kappos, 2010; Van Waesberghe et al., 1999; Van Walderveen et al., 1998). Additionally, we investigated whether exploiting the covariance between the metrics (Mangeat, Govindarajan, Mainero, & Cohen‐Adad, 2015) to generate a composite metric could lead to higher sensitivity to tissue damage than any of the individual metrics.

## 2 METHODS

### 2.1 Participants

We collected demographic, clinical, and MRI data from self‐reported right‐handed MS patients, who fulfilled the following eligibility criteria: between 18 and 60 years of age, no relapse or change in treatment for at least three months before study entry, and no other neurological or psychiatric conditions. Patients with relapsing or progressive (Lublin et al., 2014) MS (Polman et al., 2011) were recruited through the Helen Durham Centre for Neuroinflammation at the University Hospital of Wales. We used the Expanded Disability Status Scale (EDSS, Kurtzke (1983)) score and measures from the MS functional composite (MSFC, Cutter et al. (1999)) to characterise disease severity. A subsequent follow‐up, four weeks later, ensured that patients had been in a period of clinical stability during the experiment. We also recruited healthy controls to undergo the same MRI protocol.

The study was approved by the NHS South‐West Ethics and the Cardiff and Vale University Health Board R&D committees. All participants provided written informed consent.

### 2.2 MRI acquisition

MRI data were acquired on a 3T General Electric HDx MRI system (GE Medical Systems, Milwaukee, WI) using an eight channel receive‐only head RF coil (GE Medical Devices, Milwaukee, WI). The following MRI sequences were acquired: a T2/proton‐density (PD)‐weighted sequence and a fluid‐attenuated inversion recovery (FLAIR) sequence for identification and segmentation of T2‐hyperintense MS lesions; a high‐resolution T1‐weighted sequence for identification of T1‐hypointense MS lesions and for registration; a twice refocused diffusion‐weighted sequence (40 uniformly distributed directions, b = 1200 s/mm^2^), a 3D MT sequence and mcDESPOT sequences (Deoni, Rutt, Arun, et al., 2008) to obtain microstructure‐sensitive parameter maps. The acquisition parameters of all scan sequences are reported in Table 1.

**Table 1:** Scan parameters. All sequences were acquired at 3T. For each of the sequences, the main acquisition parameters are provided. The PD/−T2‐weighted and FLAIR sequences were used for semi‐automatic lesion segmentation. The DTI, MT and mcDESPOT sequences were used to obtain maps of the microstructural parameters fractional anisotropy (FA), radial diffusivity (RD), magnetisation transfer ratio (MTR), and myelin water fraction (MWF). FLAIR: fluid‐attenuated inversion recovery; FSPGR: fast spoiled gradient echo; SE: spin‐echo; IR: inversion recovery; EPI: echo‐planar imaging; EFGRE: enhanced fast gradient echo; SPGR: spoiled gradient recalled‐echo; bSSFP: balanced steady‐state free precession; IR‐SPGR: inversion recovery spoiled gradient; TE: echo time; TR: repetition time; TI: inversion time

	T1‐weighted	PD/T2‐weighted	FLAIR (T2‐weighted)	DTI	MT	mcDESPOT
Pulse sequence(s)	FSPGR	SE	Se\IR	Se\epi	EFGRE	SPGR, bSSFP, IR‐SPGR
Native resolution (mm^3^)	1.0 × 1.0 × 1.0	0.94 × 0.94 × 4.5	0.86 × 0.86 × 4.5	1.8 × 1.8 × 2.4	0.94 × 0.94 × 1.9	1.7 × 1.7 × 1.7
Field of view (mm)	256	240	220	230	240	220
Matrix size	256 × 256 × 172	256 × 256	256 × 256	96 × 96 × 36	128 × 128 × 100	128 × 128 × 88
Slices	None ‐ 3D	36 (3 mm + 1.5 mm gap)	36 (3 mm + 1.5 mm gap)	57	None ‐ 3D	None ‐3D
Total acquisition time (min)	7.5	2	3	12.5	4.5	10
TE, TR (ms)	3.0, 7.8	9.0/80.6, 3000	122.3, 9502	94.5, 16000	1.8, 26.7	SPGR: 2.1, 4.7 bSSFP: 1.6, 3.2 IR‐SPGR: 2.1, 4.7
TI (ms)	450	‐	2,250	‐	‐	IR‐SPGR: 450
Off‐resonance pulse					450°, 2 kHz off	
Flip angle (degrees)	20	90	90	90	5	SPGR: [3, 4, 5, 6, 7, 8, 9, 13, 18] bSSFP: [10.6, 14.1, 18.5, 23.8, 29.1, 35.3, 45, 60] IR‐SPGR: [5]

### 2.3 T2‐hyperintense lesion marking

MS lesions were segmented semi‐automatically using the Jim software package (v.6, Xinapse) on the T2‐weighted image, also consulting the FLAIR and the PD‐weighted images. This was done by two independent operators (IL and ES), in order to assess inter‐operator reliability of the lesion maps. We assessed the reliability in two ways: firstly, we calculated an intraclass correlation coefficient (ICC; Shrout and Fleiss (1979)) for the lesion volumes resulting from the two operators' segmentations. Secondly, to quantify the localisation agreement of the two operators' maps for each patient, we derived the Dice coefficient as a similarity index (Dice, 1945). This was calculated for each patient as twice the number of voxels marked by both operators divided by all the voxels marked by either of the operators (Zijdenbos, Member, Dawant, Margolin, & Palmer, 1994).

### 2.4 Tissue‐state segmentation

We segmented three white matter tissue‐states in each patient: **(i)** normal appearing white matter (*NAWM*); **(ii)** lesional tissue that appears as T2‐hyperintensity only (*T2L*); **(iii)** lesional tissue that shows T2‐hyperintensity as well as T1‐hypointensity (*T1L*). Average microstructural parameters were calculated for each patient within these three tissue‐states.

**Figure 1:**
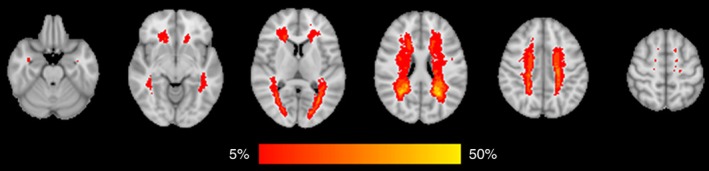
Lesion probability map. The probability map of all white matter lesions detected in all scanned 135 MS patients is shown here. The map shows voxels which were lesioned in at least 5% of the patients (the colour bar ranges from 5% to 50%). The map was used in order to restrict our analyses to white matter regions that are sensitive to the occurrence of lesions. This was done by thresholding the map at 5% and registering the resulting mask to each patient's native space [Color figure can be viewed at http://wileyonlinelibrary.com]

We restricted our regions of interest (ROIs; i.e., *NAWM*, *T2L* and *T1L*) to white matter that is more likely to show MS damage in order to minimise differences in MRI metrics simply due to spatial bias, that is, to systematic white matter differences related to spatial location (subcortical vs. periventricular) rather than disease pathology. To do this, we first created a lesion probability map from the lesion maps of all patients. We included all patients with available lesion maps, even if they were subsequently excluded from the analysis, in order for the lesion probability map to be as representative as possible of the typical location of white matter lesions. This was achieved by affinely registering the T2‐weighted images and lesion maps to the high‐resolution T1‐weighted images, using FSL FLIRT with 6° of freedom and the Correlation Ratio as cost function (Jenkinson, Bannister, Brady, & Smith, 2002). Then, the brain‐extracted (FSL BET, Smith (2002)) and lesion filled (Battaglini, Jenkinson, & Stefano, 2012) T1‐weighted images were non‐linearly normalised to the Montreal Neurological Institute (MNI) 152 template space, using ANTs SyN (Avants, Epstein, Grossman, & Gee, 2008), and the obtained warp was applied to the lesion maps. From the lesion maps in MNI space, we computed a probability map, which was then thresholded at 5% to identify white matter that is susceptible to lesions (see Figure 1). The resulting mask was then registered to each patient's individual space and used to constrain the ROIs for three segmented tissue‐states, which were constructed as explained below.

#### 2.4.1 NAWM ROI

We segmented the lesion filled T1‐weighted images using FSL FAST (Y. Zhang, Brady, & Smith, 2001) and thresholded the resulting white matter probability maps at 80% to obtain a conservative white matter mask. To create an individual map of *NAWM*, all voxels marked as lesions in the semi‐automated segmented lesion map produced by one of the operators and all voxels within 5 mm of these voxels, were removed from the individual white matter mask. This generated a *NAWM* ROI that excluded white matter in close proximity to lesions, which often shows diffuse damage (Fazekas et al., 1999; Seewann et al., 2009). Additionally, the *NAWM* ROI was restricted to lesion‐susceptible regions, as described above.

#### 2.4.2 ROIs for T2L and T1L

**Figure 2:**
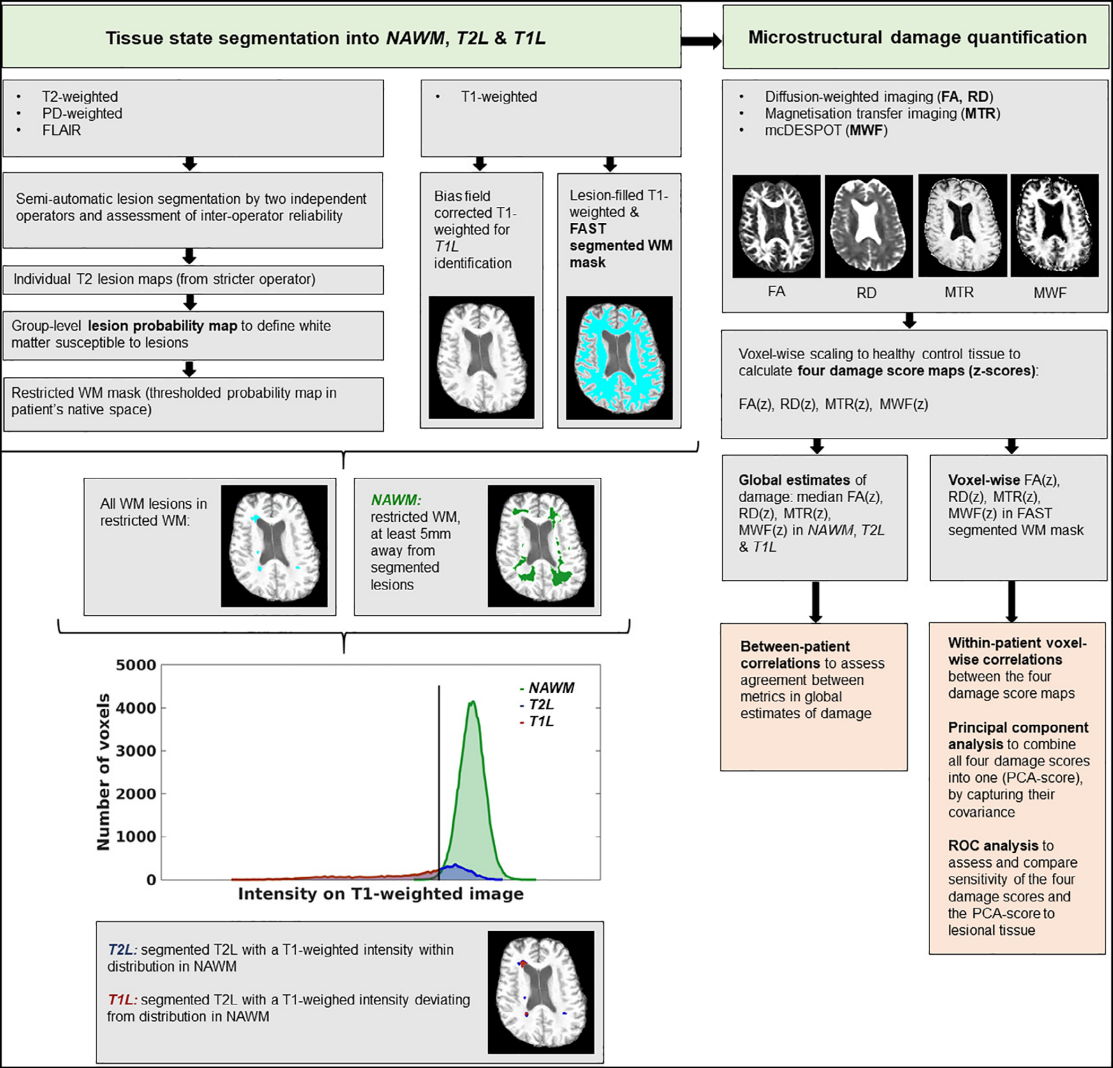
Overview of the image processing pipeline. The main analysis steps are outlined, on the left for the tissue‐state segmentation, on the right for the quantification of microstructural damage. Tissue segmentation in patients: Lesion segmentation was performed by two independent operators in order to assess reliability of the lesion segmentation. T1‐weighted images were lesion‐filled and FAST‐segmented in order to obtain a white matter mask. To restrict white matter to lesion‐susceptible regions, a lesion probability map from all patients' individual lesion maps was created and registered to each patient's native space, creating a restricted white matter mask. *NAWM* was defined as restricted white matter, at least 5 mm away from lesions in order to avoid tissue damage around lesions. White matter lesions were further segmented into *T1L* and *T2L*, based on the intensity in the (bias field corrected) T1‐weighted image. The distribution of the intensities on the T1‐weighted image in *NAWM* voxels is shown in green, while the distribution of the intensities on the T1‐weighted image in lesional voxels is shown in red and in blue. From the distribution of *NAWM* voxels, a cut‐off was calculated (1.5 IQR below the lower quartile, shown as black line) that was applied to all voxels in the lesion map. Lesional voxels with an intensity below the cut‐off were classified as *T1L* (red distribution), the rest as *T2L* (blue distribution). Microstructural damage quantification: For each patient, we derived a parameter map for each FA, RD, MTR, and MWF. We scaled these maps to the distribution (mean and *SD*) of healthy controls through z‐standardisation, yielding maps of FA(z), RD(z), MTR(z), and MWF(z), respectively. From these, global estimates of damage were obtained from the three segmented tissue‐states. Additionally, voxel‐wise values were considered within each patient's white matter mask in order to (a) look at within‐patient voxel‐wise correlations, (b) combine the four measures through a principal component analysis, and (c) assess sensitivity of each measure to lesional tissue, using a receiver operating characteristic (ROC) analysis. WM: white matter; *NAWM*: normal appearing white matter; *T2L*: T2‐hyperintense only lesional tissue; *T1L*: T2‐hyperintense lesional tissue that appears also T1‐hypointense. FA: fractional anisotropy; RD: radial diffusivity; MTR: magnetisation transfer ratio; MWF: myelin water fraction [Color figure can be viewed at http://wileyonlinelibrary.com]

We classified each T2‐hyperintense voxel into either *T2L* or *T1L*, *T1L* being T2‐hyperintense with corresponding T1‐hypointensity, and *T2L* being T2‐hyperintense without corresponding T1‐hypointensity. The classification of T2‐lesion voxels into *T2L* and *T1L* was based on the intensity on the T1‐weighted image. For each patient, we first corrected the T1‐weighted image for potential bias fields, using FAST (Y. Zhang et al., 2001). Then, we generated a distribution of image intensity values for all voxels in the *NAWM* mask. We classified each voxel in the T2‐lesion mask as *T1L* if its T1 signal intensity lay at least 1.5 interquartile ranges (IQR) below the lower quartile of the *NAWM* distribution. Visual inspection confirmed that these voxels were visibly hypointense on the T1‐weighted image. All T2‐hyperintense voxels that were not classified as *T1L* were classified as *T2‐hyperintense* only (*T2L*; Figure 2). The categories *T2L* and *T1L* are therefore mutually exclusive within a voxel. A single lesion could consist of *T2L* as well as *T1L* voxels. The *T1L* and *T2L* ROIs were restricted to lesion‐susceptible regions, as described above. Figure 2 shows the segmented tissue map for one representative MS patient.

To make comparisons between the three tissue‐states possible, in this analysis we only included participants that had tissue in all three segmented states. We established a threshold of a minimum threshold of >0.4 cm^3^ for each of the tissue‐states. This threshold roughly equals 100 voxels in the clinical scans, providing a large number of data points per included patient and tissue‐state.

### 2.5 MRI processing

#### 2.5.1 DTI: FA, RD and AD maps

The DTI data were preprocessed in ExploreDTI (v 4.8.3; Leemans, Jeurissen, Sijbers, and Jones (2009)). Data were corrected for head motion, distortions induced by the eddy currents of the diffusion‐weighted gradients and EPI‐induced geometrical distortions by registering each diffusion image to its respective (brain extracted and downsampled to 1.5 mm) T1‐weighted image (Irfanoglu, Walker, Sarlls, Marenco, & Pierpaoli, 2012) using Elastix (S. Klein, Staring, Murphy, Viergever, & Pluim, 2010), with appropriate reorientation of the diffusion‐encoding vectors (Leemans & Jones, 2009). RESTORE (L.‐C. Chang, Jones, & Pierpaoli, 2005) was used to account for outliers. FA and RD and axial diffusivity (AD) maps were exported to NIFTI format and up‐sampled to the high‐resolution structural image (1 mm isotropic).

#### 2.5.2 MTR maps

The MTR was calculated voxel by voxel with the equation MTR = [(S^0^‐S^*MT*^)/S^0^]x100, whereby S^0^ represents the signal without the off‐resonance pulse and S^*MT*^ represents the signal with the off‐resonance pulse. The MTR images in native space were skull‐stripped using FSL BET and non‐linearly registered to the respective skull‐stripped T1‐weighted images using Elastix (S. Klein et al., 2010).

#### 2.5.3 mcDESPOT and MWF maps

Spoiled gradient recalled‐echo (SPGR) and balanced steady‐state free precession (bSSFP) data sets for each participant were co‐registered to the first volume in the sequence in order to correct for inter‐scan motion using an affine (12° of freedom, mutual information) transformation (Jenkinson et al., 2002). SPGR and inversion recovery spoiled gradient (IR‐SPGR) images were used for driven equilibrium single pulse observation of T1 (DESPOT1) processing (Deoni, 2007), resulting in quantitative T1 maps. Furthermore, T2 maps were calculated with the two phase‐cycled bSSFP data using the driven equilibrium single pulse observation of T2 (DESPOT2) algorithm (Deoni, Ward, Peters, & Rutt, 2004). The data from the DESPOT1 and DESPOT2 sequences were combined according to the three‐pool multicomponent DESPOT pipeline (Deoni, Rutt, Arun, et al., 2008; Deoni, Rutt, & Jones, 2008) that yields whole brain voxel‐wise estimates of the myelin water fraction (MWF), while accounting for CSF‐partial volume contamination (Deoni, Matthews, & Kolind, 2013). MWF maps were non‐linearly registered to the brain‐extracted T1‐weighted image using Elastix (S. Klein et al., 2010).

### 2.6 Maps of microstructural damage

To scale the microstructural measures to the healthy control distribution in a voxel‐wise manner, we co‐registered all participants' maps. The brain‐extracted lesion‐filled T1‐weighted images were first non‐linearly normalised to MNI space, using ANTs SyN (Avants et al., 2008). We then applied the obtained warp to the four microstructural parameter maps, which had previously been co‐registered the T1‐weighted image, as described above. For each metric, voxel‐wise mean and *SD* maps were computed from the data sets of the healthy controls. Then, for each patient and each metric, a map was calculated showing the z‐score from the healthy control distribution for each voxel. For example, a z‐score of −1 in a patient's voxel indicated that the measure was 1 *SD* lower than the mean of the metric in healthy controls in the same location. For all further analyses, the damage scores (scaled metrics) are used and reported as FA(z), RD(z), AD(z), MTR(z) and MWF(z), respectively.

### 2.7 Global measures of damage for between‐patient correlations

To explore the agreement between the measures when estimating microstructural damage, we extracted a global damage measure for each patient and each tissue‐state. We calculated the median % z‐score from each patient's three ROIs and from each of the four metrics. We then compared the damage scores across tissue‐states using paired *t‐tests*. Then, for each tissue‐state, we computed Pearson correlation coefficients between the estimates of damage obtained from each of the four metrics.

### 2.8 Within‐patient voxel‐wise correlations

As between‐patient correlations are affected by the variability of the measures in the particular sample investigated, we also calculated voxel‐wise correlations between the metrics. This was done considering all voxels within the white matter maps of each patient. To report these correlations, we calculated the mean and *SD* of the correlation coefficient (applying z‐transformation) across the group. Additionally, we set up multiple linear regressions, each of which used a different metric as the dependent variable. We calculated the spatial variance explained in that metric by the other three metrics. This was done using in‐house software written in MATLAB (v. R2015, Mathworks).

### 2.9 Principal component analysis

To test whether combining the four considered metrics can provide a measure with increased sensitivity to lesional tissue, the covariance between the considered metrics was exploited in a principal component analysis (PCA). For this analysis, we included all voxels within all patients' white matter masks. The PCA was calculated using the MATLAB (v. R2015, Mathworks) function *PCA* over the damage scores FA(z), RD(z), MTR(z) and MWF(z). Using the resulting component weights, a component score map for the first extracted component was calculated for each participant. This component score map reflects microstructural damage as estimated by the weighted linear combination of the four metrics.

### 2.10 Sensitivity to tissue‐states using receiver operating characteristic (ROC)

To assess the sensitivity of the four metrics (FA(z), RD(z), MTR(z), MWF(z)) and of the component score from PCA to lesional tissue, for each patient, ROC analyses were conducted using the MATLAB (v. R2015, Mathworks) function *perfcurve*. ROC analyses tested the ability of the metrics to classify: (a) *T2L* versus *NAWM*; (b) *T1L* versus *NAWM*; (c) *T1L* versus *T2L*. For each ROC analysis, the area under the curve (AUC) was computed and AUCs of the metrics were statistically compared across all patients using paired *t‐tests*.

## 3 RESULTS

### 3.1 Participants' characteristics

Out of 135 scanned patients, 123 had FA, RD, MTR and MWF maps available, and were considered for the analysis. All 27 recruited healthy controls had FA, RD and MTR maps; 25 had also MWF maps. A full set of metrics could not be collected from all participants due to specific absorption rate (SAR)‐constraints of the mcDESPOT sequences or to logistical reasons.

From the 123 complete patient datasets, 105 patients met our criterion of having at least 0.4 cm^3^ volume in each of the three tissue‐states. Table 2 shows the demographic and clinical characteristics of the patients and healthy controls included in the analysis. Patients were younger than healthy controls, had smaller whole brain and grey matter volumes and performed significantly worse on tasks measuring dexterity, walking ability and cognition. None of the patients experienced a relapse or worsening of their symptoms in the four weeks after the scan.

**Table 2:** Demographic and clinical characteristics of the participants. Unless otherwise indicated, descriptive statistics provided are means and SDs. For statistical comparison between the two groups, chi‐square tests were computed for categorical variables, Kruskal‐Wallis test for skewed variables (9‐HPT, T25‐FW), and unpaired t‐tests for the rest. P values for group differences are provided. RRMS: relapsing–remitting multiple sclerosis; IQR: inter‐quartile range; EDSS: expanded disability status scale; PMS: progressive MS (primary or secondary); 9‐HPT: 9 hole peg test (score averaged across two trials); T25‐FW: timed 25 ft walk (score averaged across two trials), PASAT: paced auditory serial addition test (3 sec. Version); NBV: normalised brain volume; NGMV: normalised grey matter volume. Normalised brain and grey matter volume were calculated using SIENAX (Smith et al., 2002)

	Patients	Healthy controls	*p*‐value
n	105	27	‐
Age (years)	44.2 ± 9.1	38.1 ± 11.0	<0.01
Sex (women/men)	64/41	15/12	0.61
Education (years)	15.6 ± 4.0	20.1 ± 4.3	<0.0001
Disease duration (years)	13.1 ± 7.6	‐	‐
Disease course (RRMS/PMS)	83/22	‐	‐
Median ± IQR EDSS score	4.0 ± 1.8	‐	‐
Median ± IQR dominant (right) 9‐HPT in sec.	26.1 ± 12.8	18.9 ± 2.1	<0.01
Median ± IQR T25‐FW in sec.	5.6 ± 3.1	4.3 ± 1.1	<0.0001
PASAT (3 sec.), number of correct responses	40.7 ± 13.4	51.2 ± 6.2	<0.001
NBV (cm^3^)	1,177.8 ± 119.9	1,261.8 ± 107.0	0.0012
NGMV (cm^3^)	598.6 ± 63.3	656.7 ± 47.8	<0.0001

### 3.2 Lesion segmentation

The inter‐operator reliability for extracted lesion volume was ICC(3,1) = 0.79, *F* = 8.4, *p* < 0.0001. This indicates high agreement between lesion volumes obtained from the lesion maps segmented by the two independent operators. The mean similarity index between the two lesion maps for each patient was 0.52 ± 0.14, indicating an average of 50% spatial overlap between the lesion maps segmented by the two independent operators. Visual inspection showed that the operators differed in their conservativeness at the lesion boundaries, with one operator consistently marking more tissue than the other (*t*[134] = −6.8, *p* < 0.0001).

Beyond the exclusion of voxels in close proximity to lesions when creating the *NAWM* maps, we used also the lesion maps of the more conservative rater in order to increase confidence in the results of our tissue‐state segmentation.

The lesion probability map is shown in Figure 1. On average, patients had 62.9 ± 29.8 cm^3^ of segmented *NAWM*, 7.0 ± 6.4 cm^3^ of segmented *T2L* and 3.3 ± 3.5 cm^3^ of segmented *T1L*. As indicated above, 105 participants had at least 0.4 cm^3^ volume in each tissue‐state and were considered for all further analyses. Across the entire sample, the correlation between total lesion volume and disease severity (as measured by the MSIS‐29; *r*[133] = 0.15, *p* = 0.08), and disease duration (*r*[132] = 0.09, *p* = 0.33) were low and not significant.

### 3.3 Microstructural damage in MS lesions

**Figure 3:**
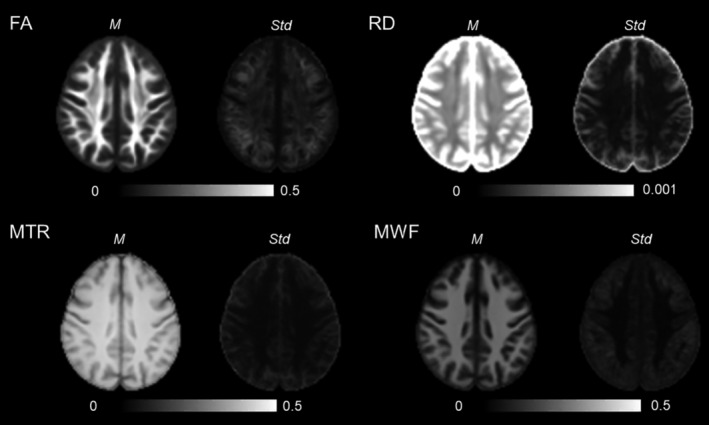
Mean and *SD* maps for the four parameters in the healthy controls. For each of the metrics, a mean (left) and *SD* (right) map is shown. The range of displayed values is adjusted to the units of the measures (0–0.5 for FA, MTR, and MWF, and 0–0.001 10^−3^ mm^2^/s for RD). The low values in the *SD* maps compared to the mean maps indicate good alignment of white matter structures across the healthy controls. The mean and *SD* maps were used to compute voxel‐wise tissue damage scores (z‐scores) in patients. Individual maps from 27 (25 for MWF) healthy controls contributed to the mean and *SD* maps. FA: fractional anisotropy; RD: radial diffusivity; MTR: magnetisation transfer ratio; MWF: myelin water fraction

Maps of mean and *SD* of the parameters in healthy controls, which were used to calculate the damage scores in patients, are shown in Figure 3. The low variability of the measures in the white matter compared to their means indicates good alignment of white matter structures after the MNI normalisation.

**Figure 4:**
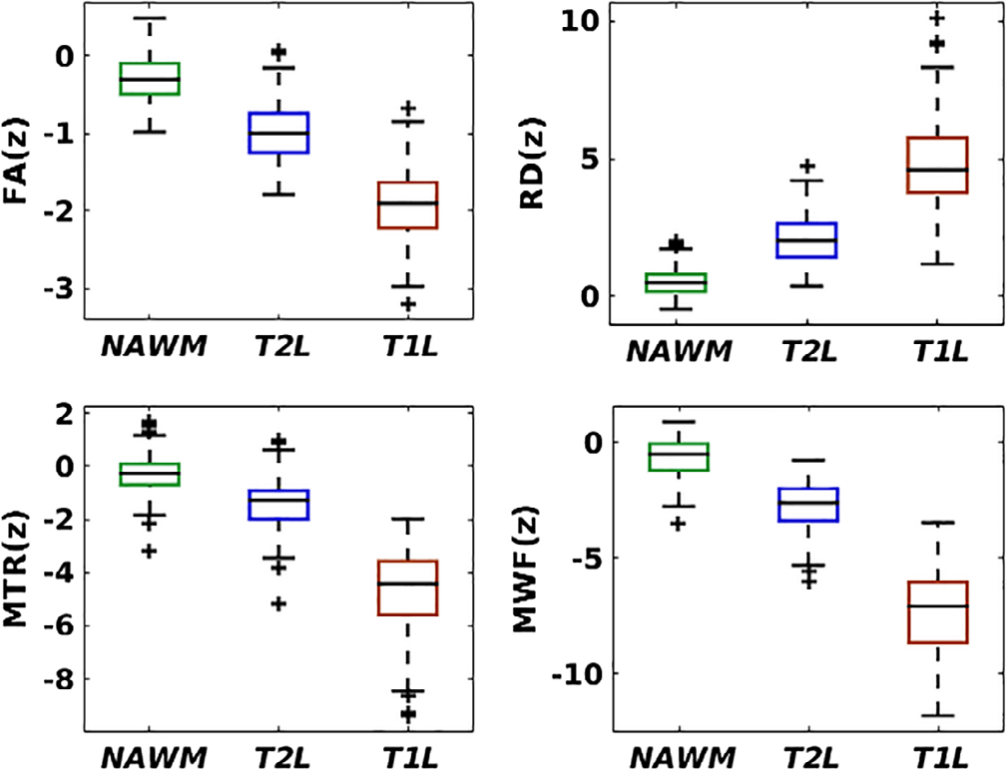
Estimates of global damage. For each metric, a boxplot across the included 105 patients is shown, comparing global damage estimates for each tissue‐state. Global damage measures were computed as the median z‐score within each tissue‐state for each patient. All differences between tissue‐states are significant for all damage scores as presented in Table 3. *NAWM*: normal appearing white matter; *T2L*: T2‐hyperintense only lesional tissue; *T1L*: T2‐hyperintense lesional tissue that appears also T1‐hypointense; FA(z): z‐score for fractional anisotropy; RD(z): z‐score for radial diffusivity; MTR(z): z‐score for magnetisation transfer ratio; MWF(z): z‐score for myelin water fraction [Color figure can be viewed at http://wileyonlinelibrary.com]

Figure 4 shows the global measures of damage for all metrics and all tissue‐states. Patients presented significant differences (*p* < 0.05) from healthy control tissue in *NAWM*, with significant decreases of FA, MTR and MWF, and significant increases in RD. Additionally, FA(z), MTR(z), MWF(z) were significantly lower in *T2L* than in *NAWM*, while RD(z) was significantly higher. Differences between *T1L* and *T2L* were also significant for all metrics. All statistical tests are reported in Table 3.

**Table 3:** Descriptive statistics for global estimates of damage. Group average (mean ± SD) for the global damage measure (average damage, computed as z‐scores, within each tissue‐state) are presented for all metrics. To compare the damage estimates across tissue‐states, t‐tests were calculated (t‐tests against 0 for the damage scores in NAWM, and paired t‐tests comparing T2L to NAWM, and T1L to T2L). Respective *t* and *p* values are reported. NAWM: normal appearing white matter; T2L: T2‐hyperintense only lesional tissue; T1L: T2‐hyperintense lesional tissue that appears also T1‐hypointense; FA(z): z‐score for fractional anisotropy; RD(z): z‐score for radial diffusivity; MTR(z): z‐score for magnetisation transfer ratio; MWF(z): z‐score for myelin water fraction

Measure	*NAWM*	*T2L*	*T1L*	*NAWM* versus 0 (*t*;*p*)	*T2L* versus *NAWM* (*t*;*p*)	*T1L* versus *T2L* (*t*;*p*)
FA(z)	−0.31 ± 0.30	−0.99 ± 0.380	−1.92 ± 0.48	−10.60; *p* < 0.001	−26.38; *p* < 0.001	−18.16; *p* < 0.001
RD(z)	0.53 ± 0.54	2.12 ± 0.884	4.84 ± 1.75	10.08; *p* < 0.001	23.00; *p* < 0.001	13.75; *p* < 0.001
MTR(z)	−0.28 ± 0.79	−1.41 ± 1.01	−4.73 ± 1.66	−3.63; *p* < 0.001	−22.46; *p* < 0.001	−13.86; *p* < 0.001
MWF(z)	−0.70 ± 0.85	−2.90 ± 1.21	−7.31 ± 1.97	−8.49; *p* < 0.001	−28.19; *p* < 0.001	−15.15; *p* < 0.001

#### 3.3.1 Accounting for age and education differences between patients and controls

The healthy controls were younger (on average, 6 years) and had higher level of education (on average, 4 years) than the patients, possibly confounding the global estimates of damage, which were based on z‐scores calculated from the healthy controls. To address this, we performed additional analyses as follows, aiming to estimate the effect that the mean age and educational difference between patients and controls may have on the microstructural metrics. First voxel‐wise regression coefficients were computed (using the AFNI [Cox, 1996] function *3dTcorr1D*) between the (z‐standardised) metrics and age / years of education in the 27 healthy controls (n = 25 for MWF). We extracted the average regression coefficient from the restricted white matter mask in MNI space and then calculated the mean change in each metric that would be expected in a cohort 6 year older. The expected age‐driven changes in (z‐standardised) metrics were −0.08 for FA(z), 0.09 for RD(z), −0.07 for MTR(z), and −0.03 for MWF(z), the expected education‐driven changes in (z‐standardised) metrics were −0.03 for FA(z), 0.02 for RD(z), −0.21 for MTR(z), and −0.10 for MWF(z) while the observed changes in *NAWM*, as reported in Table 3, were around 5 times greater (apart from for MTR): −0.30 for FA(z), 0.52 for RD(z), −0.28 for MTR(z), and  −0.70 for MWF(z). This indicates that although the age and educational difference between patients and controls is likely to have had an effect on the microstructural metrics, the effect is considerably smaller when compared to pathology‐driven changes.

### 3.4 Between‐patient correlations for global damage estimates

**Figure 5:**
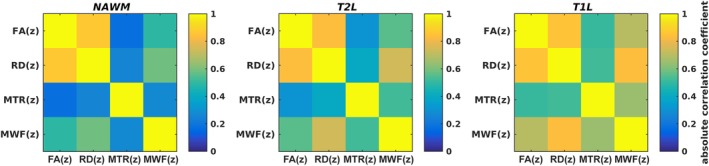
Between‐patient correlation coefficients for global damage scores. A correlation matrix is shown for each tissue‐state separately. The absolute correlation coefficient is plotted, as RD correlates negatively with the other metrics. *NAWM*: normal appearing white matter; *T2L*: T2‐hyperintense only lesional tissue; *T1L*: T2‐hyperintense lesional tissue that appears also T1‐hypointense; FA(z): z‐score for fractional anisotropy; RD(z): z‐score for radial diffusivity; MTR(z): z‐score for magnetisation transfer ratio; MWF(z): z‐score for myelin water fraction [Color figure can be viewed at http://wileyonlinelibrary.com]

**Figure 6:**
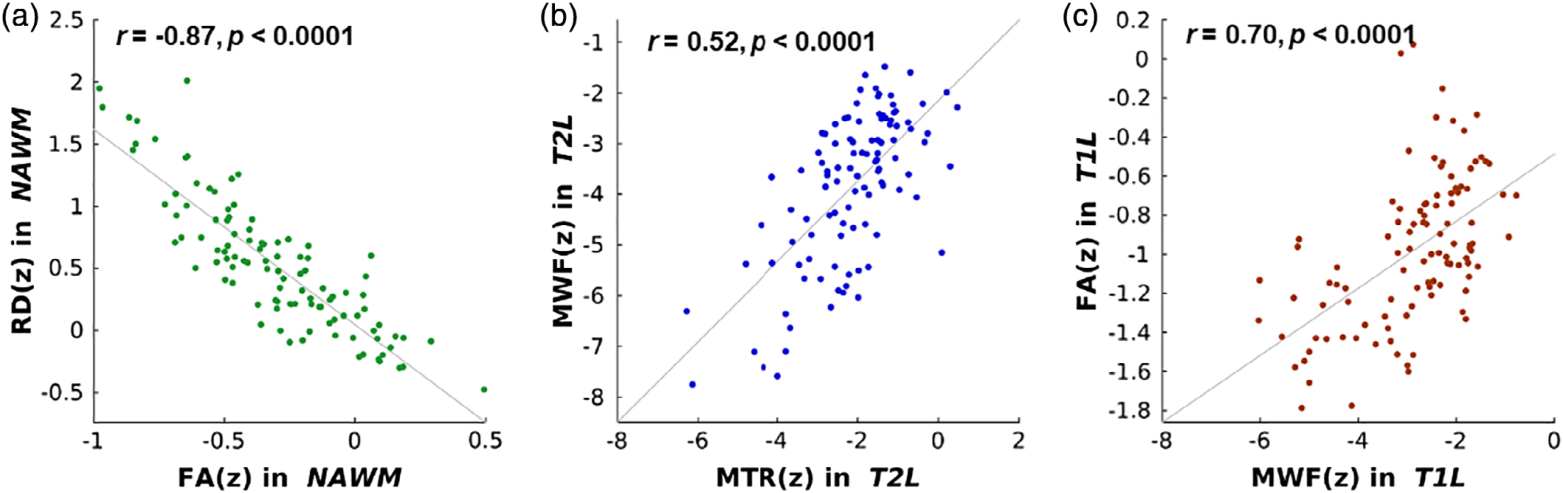
Example scatter plots for between‐patient correlations. a, Relationship between average damage in *NAWM* as estimated by FA(z) and RD(z). b, Relationship between average damage in *T2L* as estimated by MTR(z) and MWF(z). c, Relationship between damage in *T1L* as estimated by MWF(z) and FA(z). *NAWM*: normal appearing white matter; *T2L*: T2‐hyperintense only lesional tissue; *T1L*: T2‐hyperintense lesional tissue that appears also T1‐hypointense; FA(z): z‐score for fractional anisotropy; RD(z): z‐score for radial diffusivity; MTR(z): z‐score for magnetisation transfer ratio; MWF(z): z‐score for myelin water fraction [Color figure can be viewed at http://wileyonlinelibrary.com]

Correlation coefficients for global damage estimates were computed for each tissue‐state separately and showed medium‐to‐high correlations coefficients ranging from 0.18 to 0.87 between the measures. At a significance level of *p* < 0.05, all correlation coefficients were significant, apart from the one between FA with MTR in *NAWM* (*p* = 0.06; Figure 5). Applying a Bonferroni correction for multiple comparisons (corrected *p* threshold = 0.0014), most correlations were still statistically significant, the exceptions being the correlations between FA and MTR in *NAWM*, between RD and MTR in *NAWM*, between MTR and MWF in *NAWM* and between FA and MTR in *T2L*. Example scatter plots for three of the between‐patient correlations are provided in Figure 6.

### 3.5 Within‐patient voxel‐wise correlations

As for the between‐patient correlations within tissue‐averaged estimates, average (absolute) voxel‐wise correlation coefficients lay between 0.38 and 0.74, indicating medium‐to‐high spatial correlations across the maps of damage (Table 4). Additionally, we computed how much of each metric's spatial variance could be explained through a linear combination of the other metrics. On average, this was between 52% and 65% (Table 4). This indicates that maps for any of the four metrics cannot be reconstructed through a linear combination of the three other metrics, without losing around 40% of the information.

**Table 4:** Within‐patient correlations. Correlation coefficients between the four damage scores (z‐scores) across all voxels within each patient's white matter mask were calculated. Mean and SD of the correlation coefficients across patients are presented. The spatial variance in each measure explained by the other three measures is also reported (in %). FA(z): z‐score for fractional anisotropy; RD(z): z‐score for radial diffusivity; MTR(z): z‐score for magnetisation transfer ratio; MWF(z): z‐score for myelin water fraction

	FA(z)	RD(z)	MTR(z)	MWF(z)	% variance explained
**FA(z)**	1	−0.74 ± 0.08	0.38 ± 0.10	0.43 ± 0.08	56 ± 5
**RD(z)**		1	−0.52 ± 0.21	−0.58 ± 0.19	65 ± 7
**MTR(z)**			1	0.67 ± 0.23	57 ± 17
**MWF(z)**				1	52 ± 14

### 3.6 Combining all scores into one by capturing their covariance

To make component scores comparable across participants, a PCA was run for all the white matter voxels from the 105 patients by using the z‐scores from each of the four metrics as considered variables. The Kaiser‐Meyer‐Olkin index (Kaiser & Rice, 1974) of the variable matrix was 0.71, indicating that performing a PCA was reasonable. Only the first component had an eigenvalue >1 and could explain 66% of the variance. The component weights were 0.47 for FA(z), −0.55 for RD(z), 0.46 for MTR(z) and 0.51 for MWF(z). We computed a component score map for the first component, which represents microstructural damage as estimated by the combination of the four metrics. Then, using a ROC analysis, we compared this new measure (referred to as PCA score) to the four original measures with regard to its sensitivity to the three tissue‐states, as described below.

### 3.7 Comparing sensitivity of the measures to the three tissue‐states using ROC analysis

We performed a ROC analysis for each patient and metric, detecting the accuracy of that metric to classify *T2L* versus *NAWM*, *T1L* versus *NAWM*, and *T1L* versus *T2L*. We quantified the classification accuracy as the area under the resulting curve (AUC). Average AUCs ranged between 0.68 and 0.98 (Table 5), which indicates above‐chance classification performance.

**Table 5:** Area under the curve (AUC). For each metric and each classification problem, the mean ± SD AUC across patients are provided. The AUC quantifies the performance of each metric when classifying T2L versus NAWM, T1L versus NAWM, and T1L versus T2L, respectively. NAWM: normal appearing white matter; T2L: T2‐hyperintense only lesional tissue; T1L: T2‐hyperintense lesional tissue that appears also T1‐hypointense; FA(z): z‐score for fractional anisotropy; RD(z): z‐score for radial diffusivity; MTR(z): z‐score for magnetisation transfer ratio; MWF(z): z‐score for myelin water fraction; PCA‐score: score derived from the first principal component of our PCA analysis

	FA(z)	RD(z)	MTR(z)	MWF(z)	PCA‐score
*T2L* versus *NAWM*	0.68 ± 0.07	0.80 ± 0.06	0.80 ± 0.07	0.84 ± 0.05	0.83 ± 0.05
*T1L* versus *NAWM*	0.85 ± 0.06	0.94 ± 0.04	0.98 ± 0.02	0.96 ± 0.04	0.98 ± 0.02
*T1L* versus *T2L*	0.72 ± 0.06	0.80 ± 0.06	0.89 ± 0.04	0.81 ± 0.06	0.86 ± 0.04

**Figure 7:**
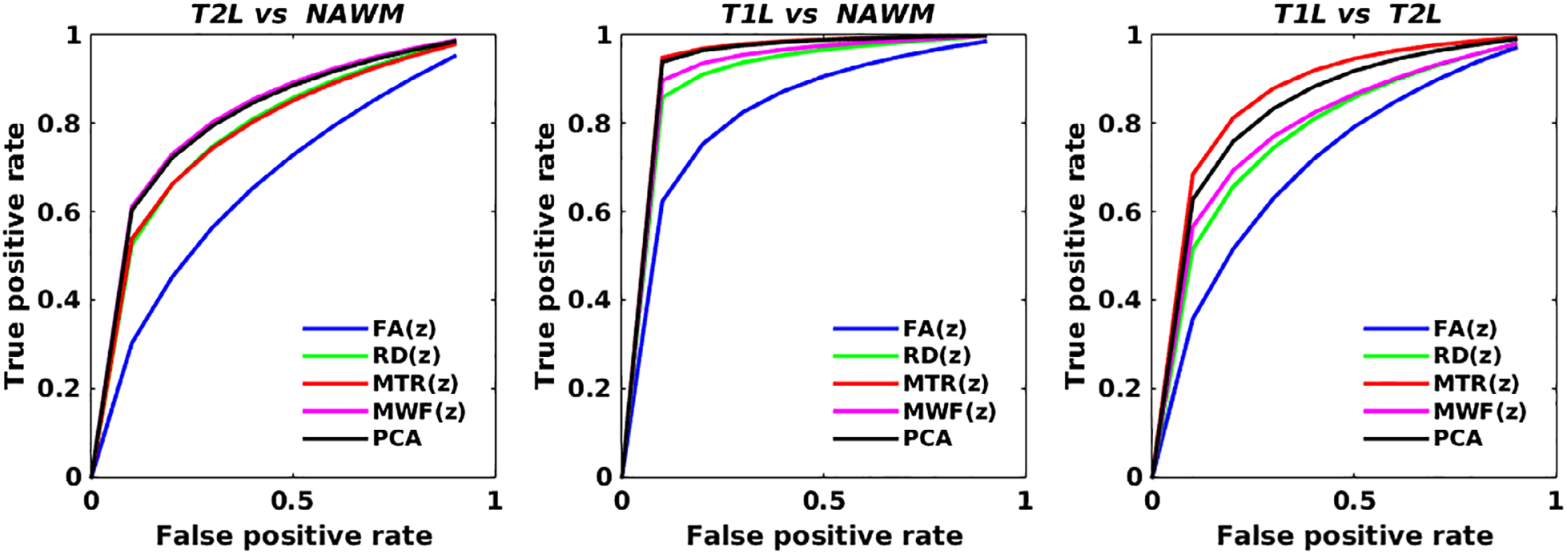
ROC analysis. For each patient, a ROC curve was computed for each classification problem: *T2L* versus *NAWM* (left plot), *T1L* versus *NAWM* (middle plot) and *T1L* versus *T2L* (right plot). The patient‐averaged ROC curve (average true positive rate depending on the set false positive rate) is plotted for each metric. To compare the performance of the metrics statistically, we considered each patient's area under the curve and performed pairwise comparisons between the metrics (as described in the text). *NAWM*: normal appearing white matter; *T2L*: T2‐hyperintense only lesional tissue; *T1L*: T2‐hyperintense lesional tissue that appears also T1‐hypointense; FA(z): z‐score for fractional anisotropy; RD(z): z‐score for radial diffusivity; MTR(z): z‐score for magnetisation transfer ratio; MWF(z): z‐score for myelin water fraction, PCA score: score derived from the first principal component of our PCA analysis [Color figure can be viewed at http://wileyonlinelibrary.com]

For the classification problem *T2L* versus *NAWM*, all pairwise comparisons between the metrics' classification performance were statistically significant when correcting for the number of tests (n = 10), apart from the comparison between RD(z) and MTR(z) (*p*[*uncorrected*] = 0.93) and between MWF(z) and the PCA score (*p*[*uncorrected*] = 0.09). The metric that could best differentiate *NAWM* from *T2L* was MWF(z), followed by the PCA score, MTR(z) and RD(z) and lastly FA(z) (Figure 7).

For the classification problem *T1L* versus *NAWM*, all pairwise comparisons were significant, apart from the comparison between MTR(z) and PCA score (*p*[*uncorrected*] = 0.20). The metrics that could best differentiate *NAWM* from *T1L* lesions were MTR(z) and the PCA score, followed by MWF(z), RD(z) and FA(z).

For the classification problem *T1L* versus *T2L*, all pairwise comparisons were significant. The metric that could best differentiate *T2L* from *T1L* lesions was MTR(z), followed by PCA score, MWF(z), RD(z) and FA(z).

## 4 DISCUSSION

Our results demonstrate that all considered metrics of white matter microstructure (FA, RD, MTR, and MWF) are sensitive to the presence and severity of MS damage. We found medium to high correlations between these metrics, with none of the metrics sharing more than 65% of their variance with the other metrics. We also showed that combining the four metrics, by capturing their covariance through a PCA, did not yield a metric more sensitive to lesional tissue than any of the individual metrics. These results suggest that, while there is some agreement between the measures of microstructural damage, they may provide complementary information on the severity of damage. Therefore, optimised clinical trials testing preventative, neuroprotective or repair intervention would benefit from the acquisition and consideration of all of these metrics to quantify the different aspects of white matter damage.

### 4.1 All four metrics indicate microstructural damage

Across the patient cohort, our four MRI‐based metrics confirmed significant microstructural damage in *NAWM*. Lesional tissue with only T2‐hyperintensity (*T2L*) showed greater damage than *NAWM* and the estimated microstructural damage was greatest in lesional tissue with additional T1‐hypointensity (*T1L*), which is consistent with histological studies (Moll et al., 2011; Sahraian et al., 2010; Van Waesberghe et al., 1999; Van Walderveen et al., 1998).

Differences in microstructural metrics between lesional tissue with and without T1‐hypointensity are not always found with MRI. For example, Vavasour, Li, Traboulsee, Moore, and Mackay (2007) reported differences between T1‐hypointense and T1‐isointense lesions only for MTR, but not for MWF. The sensitivity of MWF to different types of lesions might be influenced by the acquisition method chosen (Faizy, Thaler, Kumar, & Sedlacik, 2016), as well as by the method adopted to classify lesional tissue. In this study, instead of classifying entire lesions, we objectively classified each lesional voxel into *T2L* versus *T1L*. This maximised the contrast between the investigated tissue‐states, which was also reflected in the high classification accuracy achieved in our ROC analysis, with the average area under the curve exceeding 0.8 for all measures, apart from FA (Table 5).

### 4.2 Estimates of microstructural damage correlate between the MRI‐based measures

The global estimates of damage correlated significantly between the measures, with the lowest correlations in *NAWM*, in which the tissue damage is also the lowest. Averaging damage across lesional tissue may provide more meaningful measures than averaging across potentially more heterogeneous *NAWM*, explaining the higher between‐measure correlations found in lesional tissue. The voxel‐wise correlations that were calculated separately within each patient's white matter support the results from the between‐participant correlations of global measures of damage, also yielding medium‐to‐high correlations. This indicates that the microstructural damage maps for each patient show consistency across the metrics used.

If the covariance between the metrics were sufficiently high, this would allow the accurate generation of synthetic maps of one metric by just measuring the other metrics, potentially saving scanning time (Callaghan, Helms, Lutti, Mohammadi, & Weiskopf, 2015). However, our multiple linear regression analysis suggests that one metric cannot simply be replaced or computed from the other metrics, as on average only around 50–60% of the variance is shared between the metrics.

Overall, we showed that while spatial variance is shared between the metrics and some of the between‐patient correlations of global damage scores are high, this is not uniformly true for all metrics (e.g., the weakest between‐patient correlations were found between MTR and the other measures). This could be partly due to noise in the individual metrics and partly to the fact that each metric is sensitive to a different biological aspect of damage, which may show some independence and thus potentially reflect distinct pathological mechanisms.

### 4.3 The metrics may provide complementary information

If noise were the reason for the observed discrepancies between the metrics, then combining them into one measure could potentially provide a metric more sensitive to microstructural damage. A similar concept was implemented by Mangeat et al. (2015) with myelin‐sensitive contrasts in the cortex at 7 T: making use of the covariance structure of the metrics MT and T2*, maps of the first component score from a principal component analysis matched histology‐based cytoarchitectonic maps better than either of the two original maps.

In this study, we derived component maps from a PCA, considering all four maps of microstructural damage. The derived component score was no better than individual metrics at classifying lesional tissue from *NAWM* and *T2L* or classifying the two lesional tissue‐states. This suggests that the investigated metrics differ in their sensitivity to specific aspects of microstructural damage. The metric that was best able to discriminate between tissue‐states differed according to the classification problem. Classifying *T2L* lesions from *NAWM* was best done by MWF, while classifying *T1L* from *T2L* lesions was best done by MTR. This again suggests that the imperfect agreement between the metrics is not simply a result of noise, otherwise the least noisy metric would have the best classification power for all problems. It has to be considered that this classification analysis relies on traditional lesion maps as the gold standard to localise areas of expected histopathological damage. It is possible that the localisation of damage with the microstructural metrics and our component score is actually more accurate than operator‐dependent lesion segmentation on T1‐weighted and T2‐weighted images. Whether this is the case can only be established by the combination of histopathology and microstructural MRI.

### 4.4 MTR may be particularly sensitive to extracellular water

The particular sensitivity of MTR to *T1L* that we found supports the findings by Vavasour et al. (2007). MTR is sensitive to white matter microstructure because it quantifies the MT that happens on the surface of myelin sheaths. However, Vavasour et al. (2007) suggest that in the case of T1‐hypointense lesions, MTR changes might also reflect, to a large extent, the increase in extracellular water that follows tissue destruction. Another explanation could be that the MTR can be affected by T1‐effects (Helms, Dathe, & Dechent, 2010). By definition, T1‐hypointense lesional tissue is related to the intensity on T1‐weighted images, which could contribute to the particular sensitivity of MTR to that type of lesion. The particular sensitivity of MTR to water and T1‐effects may be the reason why it showed comparatively low between‐patient correlations with the other metrics.

### 4.5 MWF may be particularly sensitive to subtle microstructural damage in MS

MWF was significantly better than the other metrics at differentiating *T2L* from *NAWM*. MWF reflects the signal fraction coming from myelin water compared to the combined signal fraction of myelin and extracellular water. Therefore, MWF is affected by a change in the total myelin content in a voxel, but also by changes in extracellular water. *T1L* in particular is characterised by an increase in extracellular water that can follow severe tissue destruction. Nevertheless, our measure of MWF did less well than MTR at differentiating between *T1L* and *T2L*.

In the case of very severe tissue destruction, it is possible that some of the additional water is assigned to the CSF compartment rather than to the extracellular water compartment of the applied three‐compartment model. If this is the case, then the microstructural damage might be underestimated, when looking at the apparent MWF. Additional analyses show that the estimated CSF water fraction is indeed significantly increased in MS lesions, most greatly in *T1L* (Supporting Information Figure S1). MWF was particularly good at classifying lesions with less severe tissue destruction, that is, *T2L*. It could be the case that MWF provides a more specific measure than MTR in the case of less severe damage, by potentially being less affected by T1‐weighted effects. It is also possible that these two measures differ with regard to their sensitivity to fragmented myelin, which remains to be explored.

### 4.6 FA and RD are the least specific metrics

The DTI‐based measures FA and RD were the least sensitive at detecting both types of lesions. These measures are very frequently used to measure microstructural integrity of white matter, but lack specificity (Beaulieu, 2014). The diffusion tensor is sensitive to a variety of factors, including myelination and axonal density, extracellular water and fibre architecture (Beaulieu, 2014). Studies in animals suggest that axonal damage mostly affects axial diffusivity and demyelination mostly affecting perpendicular diffusivity (Concha, Gross, Wheatley, & Beaulieu, 2006; Song et al., 2003). However, in particular in MS, demyelination and axonal damage are not two isolated processes, but have a complex relationship (Simons, Misgeld, & Kerschensteiner, 2014). The microstructural damage scores we calculated using FA and RD were highly correlated, which is probably due, at least partly, to the fact that an isolated change in RD does affect FA by its definition. Still, RD had higher sensitivity to both lesional tissue‐states than FA. One explanation is that RD could be a more sensitive measure of myelination. Another explanation could be that diffusivity generally increases in lesional tissue, independent of the direction along which diffusion is measured. If this were the case, then AD, which is independent of RD, should show similar sensitivity to damage as RD. Additional analyses show that, in our data (Supporting Information Figure S2), this is not the case and that AD performs significantly worse than RD at classifying the tissue‐states, although it performs marginally better than FA. This suggests that RD may indeed be the most sensitive of the DTI‐based metrics, when estimating microstructural damage.

From the four investigated metrics, FA was the least sensitive to lesional tissue. FA is predominantly determined by structured axonal membranes (Beaulieu, 2014), but is also sensitive to changes in myelination. However, large changes in myelination lead to comparatively small changes in anisotropy (Song et al., 2002). The microstructural damage that occurs in lesional tissue is a combination of myelin and axonal loss, and FA might preferentially reflect changes in the axonal density. On the other hand, the other metrics are not only sensitive to the loss of myelinated axons, but also to demyelination in intact axons, which could explain greater estimated damage in lesional tissue.

Due to the simplicity of the diffusion tensor, DTI has limited capabilities to infer specific microstructural changes. It is a method sensitive to a variety of changes, however, and still frequently used in the clinical research in MS (Barkhof et al., 2009; Enzinger et al., 2015; Moccia, Stefano, & Barkhof, 2017; Tomassini et al., 2012). Multi‐shell diffusion acquisitions allow to apply models such as the neurite orientation dispersion and density imaging (NODDI; H. Zhang, Schneider, Wheeler‐Kingshott, and Alexander (2012)) or composite hindered and restricted model of diffusion (CHARMED; Assaf and Basser (2005)), and infer more biologically specific microstructural measures, such as the fibre orientation dispersion. Some of these metrics may have the potential to provide even more specific information about pathological processes (Tomassini et al., 2012). However, it is important to mention that due to the complexity of these models, various assumptions and model constraints have to be established. While these may be reasonable for healthy tissue, they do not necessarily hold for pathological tissue, which can make the interpretation of these microstructural metrics challenging (Lampinen et al., 2017).

The investigated metrics are likely to be sensitive to different pathological processes. Therefore, the collection of several, complementary metrics could improve detection and quantification of microstructural pathology, with important implications for clinical trials, whose behavioural or pharmacological interventions could interfere with or induce microstructural changes.

### 4.7 Limitations

The study is not without limitations. We used lesional tissue to assess the sensitivity of MRI‐based metrics to microstructural damage, as pathology is most pronounced in lesions (Tomassini & Palace, 2009). While axonal loss and demyelination are established pathological processes in MS, lesional tissue is also characterised by inflammation, gliosis, axonal swelling and edema (Filippi et al., 2012). The microstructural measures applied in this study are likely to be sensitive to some of these processes. Additionally, lesional tissue can be very heterogeneous between and within patients, with numerous types of MS lesions described histologically (Kuhlmann et al., 2017). As we cannot differentiate between these lesion types using MRI, we classified the lesions broadly into two lesional tissue‐states.

In this study, we focused on T2‐weighted white matter hyperintense lesional tissue without versus with T1‐weighted hypointensity. The majority of T1‐hypointense lesions are chronic black holes, that is, lesions with severe tissue destruction (Sahraian et al., 2010; Tomassini & Palace, 2009). However, a minority of them are active lesions, which can be distinguished on a post‐contrast T1‐weighted scan by their gadolinium‐enhancement. Ours were research scans, acquired without gadolinium‐contrast administration, which prevented us from making this distinction. However, it has to be noted that the classification of enhancing versus non‐enhancing lesions is also not straightforward, since there are a number of methodological factors that determine whether lesions enhance or not (Filippi, 2000). However, from the low proportion of active lesions among T1‐hypointense lesions reported in the literature (Ciccarelli et al., 1999; Koudriavtseva et al., 1997; Zinadinov & Bakshi, 2004), and from the inclusion criteria in our study we expect only a minority of *T1L* assessed to be enhancing lesions.

To ensure informativeness of the results, we only included patients with a high number of voxels within each tissue class. Although this could bias the sample towards patients with higher total lesion volume, we showed that this was unrelated to disease severity and duration.

When comparing the sensitivity of the metrics to lesional tissue, the ROC analysis showed AUCs between 0.68 and 0.98, which overall indicated high classification accuracy. This analysis assumes that the T2‐lesion masks are the gold‐standard. However, lesion segmentation is not a trivial process (Carass et al., 2017). As shown in our inter‐operator reliability analysis, this is highly operator‐dependent. Even if the correlation between lesion volumes calculated from two operators suggests reliable lesion segmentation, some operators can be more conservative than others (Carass et al., 2017).

The patient cohort in this study was on average 6 years older than the controls and had on average 4 years fewer of education. Our analyses show that this difference had an effect on the normalised damage scores in the patients. However, the effect size was small compared to the effect of pathology, apart from the relationship between education and MTR. However, here, causality cannot be inferred, as the disease severity may be a confound, affecting both microstructure and how long patients can stay in education for. Additionally, since all patients' maps were normalised in the same way, a potential bias in the z‐scores would not have affected the correlations between the metrics.

The high sensitivity of DTI‐based metrics to the underlying fibre configuration could contribute to explain, at least in part, their low sensitivity to lesional tissue. We aimed to address this inherent limitation of DTI by spatially normalising the metrics to healthy control tissue. However, the co‐registration of the microstructural images, which is based on T1‐weighted structural images, limits the extent to which this analysis can succeed. To minimise potential registration biases due to atrophy in our patient group, we employed ANTs SyN algorithm, which is considered as a robust method to register atrophic brains (Avants et al., 2008; A. Klein et al., 2009). Additionally, in advance of applying this method to our patient cohort, we compared it to other methods (linear and nonlinear registration provided by FSLs FLIRT and FNIRT) and investigated the effect of atrophy on the resulting MNI normalisation (Supporting Informtion Figure S3). Our results suggested that ANTs SyN provided the best registration success in our dataset, with differences between patients with higher versus lower atrophy being negligibly small compared to differences between the compared registration algorithms.

### 4.8 Conclusions

The four investigated metrics of white matter damage (FA(z), RD(z), MTR(z) and MWF(z)) show good agreement, when estimating microstructural damage in MS. Differences between the damage estimates are likely to be a result of differences in sensitivity to different aspects of pathology. These metrics, therefore, provide complementary information about microstructural damage. Considering them in combination in MS, as well as in other conditions affecting the integrity of white matter (Mole et al., 2016), can improve our understanding of tissue pathology and may offer more accurate measurement of the effect of novel therapeutic interventions for prevention, neuroprotection or repair.

## Supporting information


**Fig. 1 Changes in CSF volume fraction (CSFV)**. A boxplot across the included 105 patients is shown for global normalised (median z‐score) CSF volume fraction within each tissue‐state for each patient. Mean z‐scores are 0.15 ± 0.34 for *NAWM*, 1.16 ± 0.77 for *T2L*, and 1.93 ± 4.48 for *T1L*. In *NAWM* CSF volume fraction was significantly different from 0 (*t* = 4.5, *p* < 0.0001), while it was significantly higher in *T2L* than in *NAWM* (*t* = 18.1, *p* < 0.0001). In *T1L* CSF volume fraction was significantly higher than in *T2L* (*t* = −13.3, *p* < 0.0001). **Acronyms:** *NAWM* = normal appearing white matter, *T2L* = T2‐hyperintense only lesional tissue, *T1L* = T2‐hyperintense lesional tissue that appears also T1‐hypointense.
**Fig. 2** ROC **analysis for DTI‐based metrics.** For each patient, a ROC curve was computed for each classification problem: *T2L* vs *NAWM* (left plot), *T1L* vs *NAWM* (middle plot) and *T1L* vs *T2L* (right plot). The patient‐averaged ROC curve (average true positive rate depending on the fixed false positive rate) is plotted for each metric. For the classification problem *T2L* vs *NAWM*, on average RD performed significantly better than AD (*p* < 0.0001) or FA (*p* = 0.02). AD performed significantly better than FA (*p* = 0.02) or RD (*p* < 0.0001). For the classification problem *T1L* vs *NAWM*, on average RD performed significantly better than FA (*p* < 0.0001) and AD (*p* < 0.0001), with no performance differences between FA and AD (*p* = 0.12). For the classification problem *T1L* vs *T2L*, on average RD performed significantly better than FA (*p* < 0.0001) and AD (*p* < 0.0001). AD performed significantly better than FA (*p* = 0.01). **Acronyms:** *NAWM* = normal appearing white matter, *T2L* = T2‐hyperintense only lesional tissue, *T1L* = T2‐hyperintense lesional tissue that appears also T1‐hypointense, FA(z) = z‐score for fractional anisotropy, RD(z) = z‐score for radial diffusivity, MTR(z) = z‐score for magnetisation transfer ratio, MWF(z) = z‐score for myelin water fraction, AD(z) = z‐score for axial diffusivity.
**Fig. 3** Gro**up‐level evaluation of the effects of atrophy and registration algorithm on the normalisation to MNI space. A)** Based on the distribution of normalised grey matter volume (SIENAX, Smith et al., 2002), we split our cohort of patients (N = 134; red line) in those with lower versus higher atrophy. The threshold was established based on the 5th percentile (yellow line) of the normalised grey matter volume distribution in our healthy control cohort (N = 27; green line). **B)** We compared three algorithms linear registration (FSL FLIRT), nonlinear registration (FSL FNIRT) and diffeomorphic registration (ANTs SyN). For each algorithm and each group of patients (healthy controls, patients with lower atrophy, and patients with higher atrophy), we normalised the (lesion filled) T1‐weighted images to MNI space and averaged the resulting images across the group. The smoothness across the averaged brain provides an indication of registration success, with the worst alignment resulting in the blurriest average image, and the best alignment resulting in the clearest average image. Visual inspection of the output suggests that FLIRT resulted consistently in the blurriest images and ANTs SyN registration provided the clearest average brains. In comparison to the effects of normalisation method, the effect of atrophy on the average image seems negligible. The visual inspection of the images is corroborated by quantification of the FWHM using AFNIs *3dFWHMx* function, showing highest blurriness for FLIRT (HC: 10.8, lower atrophy: 12.6, higher atrophy: 13.2), less blurriness for FNIRT (HC: 10.5, lower atrophy: 10.4, higher atrophy: 10.3) and least blurriness for ANTs (HC: 7.1, lower atrophy: 8.5, higher atrophy: 8.7).

## References

[hbm24568-bib-0001] Assaf, Y. , & Basser, P. J. (2005). Composite hindered and restricted model of diffusion (CHARMED) MR imaging of the human brain. NeuroImage, 27(1), 48–58. 10.1016/j.neuroimage.2005.03.042 15979342

[hbm24568-bib-0002] Avants, B. B. , Epstein, C. , Grossman, M. , & Gee, J. (2008). Symmetric diffeomorphic image registration with crosscorrelation: Evaluating automated labeling of elderly and neurodegenerative brain. Medical Image Analysis, 12, 26–41. 10.1016/j.media.2007.06.004 17659998 PMC2276735

[hbm24568-bib-0003] Barkhof, F. (1999). MRI in multiple sclerosis: Correlation with expanded disability status scale (EDSS). Multiple Sclerosis, 5(4), 283–286. 10.1191/135245899678846221 10467389

[hbm24568-bib-0004] Barkhof, F. (2002). The clinico‐radiological paradox in multiple sclerosis revisited. Current Opinion in Neurology, 15(3), 239–245. 10.1097/00019052-200206000-00003 12045719

[hbm24568-bib-0005] Barkhof, F. , Calabresi, P. , Miller, D. , & Reingold, S. (2009). Imaging outcomes for neuroprotection and repair in multiple sclerosis trials. Nature Reviews Neurology, 5, 256–266. 10.1038/nrneurol.2009.41 19488083

[hbm24568-bib-0006] Basser, P. , Mattiello, J. , & LeBihan, D. (1994). MR diffusion tensor spectroscopy and imaging. Biophysical Journal, 66(1), 259–267. 10.1016/S0006-3495(94)80775-1 8130344 PMC1275686

[hbm24568-bib-0007] Battaglini, M. , Jenkinson, M. , & Stefano, N. D. (2012). Evaluating and reducing the impact of white matter lesions on brain volume measurements. Human Brain Mapping, 33(9), 2062–2071. 10.1002/hbm.21344 21882300 PMC6870255

[hbm24568-bib-0008] Beaulieu, C. (2002). The basis of anisotropic water diffusion in the nervous system—A technical review. NMR in Biomedicine, 15(7–8), 435–455. 10.1002/nbm.782 12489094

[hbm24568-bib-0009] Beaulieu, C. (2014). The biological basis of diffusion anisotropy In Johansen‐Berg H. & Behrens T. (Eds.), Diffusion MRI (2nd ed., pp. 155–184). Cambridge, Massachusetts: Academic press.

[hbm24568-bib-0010] Bells, S. , Morris, D. , & Vidarsson, L. (2007). Comparison of linear combination filtering to DTI and MTR in whole brain myelin‐water imaging. Proceedings of the International Society for Magnetic Resonance in Medicine, 15.

[hbm24568-bib-0011] Bitsch, A. , Schuchardt, J. , Bunkowski, S. , Kuhlmann, T. , & Bru, W. (2000). Acute axonal injury in multiple sclerosis: Correlation with demyelination and inflammation. Brain, 123, 1174–1183. 10825356 10.1093/brain/123.6.1174

[hbm24568-bib-0012] Callaghan, M. F. , Helms, G. , Lutti, A. , Mohammadi, S. , & Weiskopf, N. (2015). A general linear relaxometry model of R1 using imaging data. Magnetic Resonance in Medicine, 73, 1309–1314. 10.1002/mrm.25210 24700606 PMC4359013

[hbm24568-bib-0013] Carass, A. , Roy, S. , Jog, A. , Cuzzocreo, J. L. , Magrath, E. , et al. (2017). Longitudinal multiple sclerosis lesion segmentation: Resource and challenge. NeuroImage, 148, 77–102. 28087490 10.1016/j.neuroimage.2016.12.064 PMC5344762

[hbm24568-bib-0014] Chang, E. H. , Argyelan, M. , Aggarwal, M. , Chandon, T.‐S. S. , Karlsgodt, K. H. , Mori, S. , & Malhotra, A. K. (2017). Diffusion tensor imaging measures of white matter compared to myelin basic protein immunofluorescence in tissue cleared intact brains. Data in Brief, 10, 438–443. 10.1016/j.dib.2016.12.018 28054004 PMC5198630

[hbm24568-bib-0015] Chang, L.‐C. , Jones, D. K. , & Pierpaoli, C. (2005). RESTORE: Robust estimation of tensors by outlier rejection. Magnetic Resonance in Medicine, 53, 1088–1095. 10.1002/mrm.20426 15844157

[hbm24568-bib-0016] Ciccarelli, O. , Giugni, E. , Paolillo, A. , Mainero, C. , Gasperini, C. , Bastianello, S. , & Pozzilli, C. (1999). Magnetic resonance outcome of new enhancing lesions in patients with relapsing‐remitting multiple sclerosis. European Journal of Neurology, 6, 455–459. 10362899 10.1046/j.1468-1331.1999.640455.x

[hbm24568-bib-0017] Concha, L. , Gross, D. W. , Wheatley, B. M. , & Beaulieu, C. (2006). Diffusion tensor imaging of time‐dependent axonal and myelin degradation after corpus callosotomy in epilepsy patients. NeuroImage, 32(3), 1090–1099. 10.1016/j.neuroimage.2006.04.187 16765064

[hbm24568-bib-0018] Cox, R. W. (1996). AFNI: Software for analysis and visualization of functional magnetic resonance neuroimages. Computers and Biomedical Research, 29(3), 162–173. 8812068 10.1006/cbmr.1996.0014

[hbm24568-bib-0019] Cutter, G. R. , Baier, M. L. , Rudick, R. A. , Cookfair, D. L. , Fischer, J. S. , Petkau, J. , … Reingold, S. (1999). Development of a multiple sclerosis functional composite as a clinical trial outcome measure. Brain, 122, 871–882. 10355672 10.1093/brain/122.5.871

[hbm24568-bib-0020] De Santis, S. , Drakesmith, M. , Bells, S. , Assaf, Y. , & Jones, D. K. (2014). Why diffusion tensor MRI does well only some of the time: Variance and covariance of white matter tissue microstructure attributes in the living human brain. NeuroImage, 89, 35–44. 10.1016/j.neuroimage.2013.12.003 24342225 PMC3988851

[hbm24568-bib-0021] Deoni, S. C. L. (2007). High‐resolution T1 mapping of the brain at 3T with driven equilibrium single pulse observation of T1 with high‐speed incorporation of RF field. Journal of Magnetic Resonance Imaging, 26, 1106–1111. 10.1002/jmri.21130 17896356

[hbm24568-bib-0022] Deoni, S. C. L. , Matthews, L. , & Kolind, S. H. (2013). One component? Two components? Three? The effect of including a nonexchanging “free” water component in multicomponent driven equilibrium single pulse observation of T1 and T2. Magnetic Resonance in Medicine, 70(1), 147–154. 10.1002/mrm.24429 22915316 PMC3711852

[hbm24568-bib-0023] Deoni, S. C. L. , Rutt, B. K. , Arun, T. , Pierpaoli, C. , & Jones, D. K. (2008). Gleaning multicomponent T1 and T2 information from steady‐state imaging data. Magnetic Resonance in Medicine, 60(6), 1372–1387. 10.1002/mrm.21704 19025904

[hbm24568-bib-0024] Deoni, S. C. L. , Rutt, B. K. , & Jones, D. K. (2008). Investigating exchange and multicomponent relaxation in fullybalanced steady‐state free precession imaging. Journal of Magnetic Resonance Imaging, 27, 1421–1429. 10.1002/jmri.21079 18504765

[hbm24568-bib-0025] Deoni, S. C. L. , Ward, H. A. , Peters, T. M. , & Rutt, B. K. (2004). Rapid T2 estimation with phase‐cycled variable nutation steady‐state free precession. Magnetic Resonance in Medicine, 52(2), 435–439. 10.1002/mrm.20159 15282830

[hbm24568-bib-0026] Dice, L. R. (1945). Measures of the amount of ecologic association between species. Ecology, 26(3), 297–302.

[hbm24568-bib-0027] Enzinger, C. , Barkhof, F. , Ciccarelli, O. , Filippi, M. , Kappos, L. , Rocca, M. A. , … Vrenken, H. (2015). Nonconventional MRI and microstructural cerebral changes in multiple sclerosis. Nature Reviews Neurology, 11(12), 676–686. 10.1038/nrneurol.2015.194 26526531

[hbm24568-bib-0028] Faizy, T. D. , Thaler, C. , Kumar, D. , & Sedlacik, J. (2016). Heterogeneity of multiple sclerosis lesions in multislice myelin water imaging. PLoS One, 11, e0151496 10.1371/journal.pone.0151496 26990645 PMC4798764

[hbm24568-bib-0029] Fazekas, F. , Barkhof, F. , Filippi, M. , Grossman, R. I. , Li, D. K. B. , & Mcdonald, W. I. (1999). The contribution of magnetic resonance imaging to the diagnosis of. Neurology, 53, 448–456. 10449103 10.1212/wnl.53.3.448

[hbm24568-bib-0030] Filippi, M. (2000). Enhanced magnetic resonance imaging in multiple sclerosis. Multiple Sclerosis, 6(5), 320–326. 10.1177/135245850000600505 11064441

[hbm24568-bib-0031] Filippi, M. (2001). Linking structural, metabolic and functional changes in multiple sclerosis. European Journal of Neurology, 8(4), 291–297. 10.1046/j.1468-1331.2001.00210.x 11422425

[hbm24568-bib-0032] Filippi, M. , Preziosa, P. , & Rocca, M. A. (2017). Microstructural MR imaging techniques in multiple sclerosis. Neuroimaging Clinics of NA, 27(2), 313–333. 10.1016/j.nic.2016.12.004 28391789

[hbm24568-bib-0033] Filippi, M. , Rocca, M. A. , Barkhof, F. , Brück, W. , Chen, J. T. , Comi, G. , … Lassmann, H. (2012). Association between pathological and MRI findings in multiple sclerosis. The Lancet Neurology, 11(4), 349–360. 10.1016/S1474-4422(12)70003-0 22441196

[hbm24568-bib-0034] Gareau, P. J. , Rutt, B. K. , Karlik, S. J. , & Mitchell, J. R. (2000). Magnetization transfer and multicomponent T2 relaxation measurements with histopathologic correlation in an experimental model of MS. Journal of Magnetic Resonance Imaging, 11(6), 586–595. 10862056 10.1002/1522-2586(200006)11:6<586::aid-jmri3>3.0.co;2-v

[hbm24568-bib-0035] Giorgio, A. , Palace, J. , Johansen‐Berg, H. , Smith, S. M. , Ropele, S. , Fuchs, S. , … Fazekas, F. (2010). Relationships of brain white matter microstructure with clinical and MR measures in relapsing‐ remitting multiple sclerosis. Journal of Magnetic Resonance Imaging, 316, 309–316. 10.1002/jmri.22062 PMC7610900 20099343

[hbm24568-bib-0036] Helms, G. , Dathe, H. , & Dechent, P. (2010). Modeling the influence of TR and excitation flip angle on the magnetization transfer ratio ( MTR ) in human brain obtained from 3D spoiled gradient echo MRI. Magnetic Resonance in Medicine, 64, 177–185. 10.1002/mrm.22379 20572139

[hbm24568-bib-0037] Irfanoglu, M. O. , Walker, L. , Sarlls, J. , Marenco, S. , & Pierpaoli, C. (2012). Effects of image distortions originating from susceptibility variations and concomitant fields on diffusion MRI tractography results. NeuroImage, 61(1), 275–288. 10.1016/j.neuroimage.2012.02.054 22401760 PMC3653420

[hbm24568-bib-0038] Jenkinson, M. , Bannister, P. , Brady, M. , & Smith, S. (2002). Improved optimization for the robust and accurate linear registration and motion correction of brain images. NeuroImage, 17(2), 825–841. 10.1006/nimg.2002.1132 12377157

[hbm24568-bib-0039] Kaiser, H. , & Rice, J. (1974). Little Jiffy, Mark IV. Educational and Psychological Measurement, 34, 111–117.

[hbm24568-bib-0040] Kitzler, H. H. , Su, J. , Zeineh, M. , Harper‐little, C. , Leung, A. , Kremenchutzky, M. , … Rutt, B. K. (2012). Deficient MWF mapping in multiple sclerosis using 3D whole‐brain multi‐component relaxation MRI. NeuroImage, 59(3), 2670–2677. 10.1016/j.neuroimage.2011.08.052 21920444 PMC3673309

[hbm24568-bib-0041] Klawiter, E. C. , Schmidt, R. E. , Trinkaus, K. , Liang, H. F. , Budde, M. D. , Naismith, R. T. , … Benzinger, T. L. (2011). Radial diffusivity predicts demyelination in ex vivo multiple sclerosis spinal cords. NeuroImage, 55(4), 1454–1460. 10.1016/j.neuroimage.2011.01.007 21238597 PMC3062747

[hbm24568-bib-0042] Klein, A. , Andersson, J. , Ardekani, B. A. , Ashburner, J. , Avants, B. , Chiang, M.‐c. , … Parsey, R. V. (2009). Evaluation of 14 nonlinear deformation algorithms applied to human brain MRI registration. NeuroImage, 46(3), 786–802. 10.1016/j.neuroimage.2008.12.037 19195496 PMC2747506

[hbm24568-bib-0043] Klein, S. , Staring, M. , Murphy, K. , Viergever, M. A. , & Pluim, J. P. W. (2010). Elastix: A toolbox for intensity‐based medical image registration. IEEE Transactions on Medical Imaging, 29(1), 196–205. 19923044 10.1109/TMI.2009.2035616

[hbm24568-bib-0044] Kolind, S. , Matthews, L. , Johansen‐Berg, H. , Leite, M. I. , Williams, S. C. R. , Deoni, S. , & Palace, J. (2012). Myelin water imaging reflects clinical variability in multiple sclerosis. NeuroImage, 60(1), 263–270. 10.1016/j.neuroimage.2011.11.070 22155325 PMC3671155

[hbm24568-bib-0045] Kolind, S. H. , Laule, C. , Vavasour, I. M. , Li, D. K. B. , Traboulsee, A. L. , Mädler, B. , … Mackay, A. L. (2008). Complementary information from multi‐exponential T2 relaxation and diffusion tensor imaging reveals differences between multiple sclerosis lesions. NeuroImage, 40, 77–85. 10.1016/j.neuroimage.2007.11.033 18226549

[hbm24568-bib-0046] Koudriavtseva, T. , Thompson, A. J. , Fiorelli, M. , Gasperini, C. , Bastianello, S. , Bozzao, A. , … Pozzilli, C. (1997). Gadolinium enhanced MRI predicts clinical and MRI disease activity in relapsing‐remitting multiple sclerosis. Journal of Neurology, Neurosurgery, and Psychiatry, 62(3), 285–287. 10.1136/jnnp.62.3.285 PMC1064162 9069488

[hbm24568-bib-0047] Kuhlmann, T. , Ludwin, S. , Prat, A. , Antel, J. , Brück, W. , & Lassmann, H. (2017). An updated histological classification system for multiple sclerosis lesions. Acta Neuropathologica, 133, 13–24. 10.1007/s00401-016-1653-y 27988845

[hbm24568-bib-0048] Kurtzke, J. F. (1983). Rating neurologic impairment in multiple sclerosis: An expanded disability status scale (EDSS). Neurology, 33(November), 1444–1453. 6685237 10.1212/wnl.33.11.1444

[hbm24568-bib-0049] Lampinen, B. , Szczepankiewicz, F. , Mårtensson, J. , Westen, D. V. , Sundgren, C. , & Nilsson, M. (2017). Neurite density imaging versus imaging of microscopic anisotropy in diffusion MRI: A model comparison using spherical tensor encoding. NeuroImage, 147, 517–531. 10.1016/j.neuroimage.2016.11.053 27903438

[hbm24568-bib-0050] Laule, C. , Kozlowski, P. , Leung, E. , Li, D. K. B. , Mackay, A. L. , & Moore, G. R. W. (2008). Myelin water imaging of multiple sclerosis at 7 T: Correlations with histopathology. NeuroImage, 40, 1575–1580. 10.1016/j.neuroimage.2007.12.008 18321730

[hbm24568-bib-0051] Laule, C. , Leung, E. , Traboulsee, A. L. , Paty, D. W. , & Mackay, A. L. (2006). Myelin water imaging in multiple sclerosis: Quantitative correlations with histopathology. Multiple Sclerosis., 12, 747–753. 10.1177/1352458506070928 17263002

[hbm24568-bib-0052] Leemans, A. , Jeurissen, B. , Sijbers, J. , & Jones, D. (2009). ExploreDTI: A graphical toolbox for processing, analyzing, and visualizing diffusion MR data. Proceedings of the International Society for Magnetic Resonance in Medicine, 17, 3537.

[hbm24568-bib-0053] Leemans, A. , & Jones, D. K. (2009). The B‐matrix must be rotated when correcting for subject motion in DTI data. Magnetic Resonance in Medicine, 1349, 1336–1349. 10.1002/mrm.21890 19319973

[hbm24568-bib-0054] Lublin, F. D. , Reingold, S. C. , Cohen, J. A. , Cutter, G. R. , Thompson, A. J. , Wolinsky, J. S. , … Sormani, M. P. (2014). Defining the clinical course of multiple sclerosis the 2013 revisions. Neurology, 83, 278–286. 24871874 10.1212/WNL.0000000000000560 PMC4117366

[hbm24568-bib-0055] Mackay, A. , Whittall, K. , Adler, J. , Li, D. , Paty, D. , & Graeb, D. (1994). In vivo visualization of myelin water in brain by magnetic resonance. Magnetic Resonance in Medicine, 31(6), 673–677. 10.1002/mrm.1910310614 8057820

[hbm24568-bib-0056] Mädler, B. , Drabycz, S. A. , Kolind, S. H. , Whittall, K. P. , & Mackay, A. L. (2008). Is diffusion anisotropy an accurate monitor of myelination? Correlation of multicomponent T2 relaxation and diffusion tensor anisotropy in human brain. Magnetic Resonance Imaging, 26, 874–888. 10.1016/j.mri.2008.01.047 18524521

[hbm24568-bib-0057] Mallik, S. , Samson, R. S. , Wheeler‐Kingshott, C. A. M. , & Miller, D. H. (2014). Imaging outcomes for trials of remyelination in multiple sclerosis. Journal of Neurology, Neurosurgery, and Psychiatry, 85, 1396–1404. 10.1136/jnnp-2014-307650 PMC4335693 24769473

[hbm24568-bib-0058] Mangeat, G. , Govindarajan, S. T. , Mainero, C. , & Cohen‐Adad, J. (2015). Multivariate combination of magnetization transfer, T2* and B0 orientation to study the myelo‐architecture of the in vivo human cortex. NeuroImage, 119, 89–102. 10.1016/j.neuroimage.2015.06.033 26095090 PMC4564318

[hbm24568-bib-0059] Moccia, M. , Stefano, N. D. , & Barkhof, F. (2017). Imaging outcome measures for progressive multiple sclerosis trials. Multiple Sclerosis Journal, 23(12), 1614–1626. 10.1177/1352458517729456 29041865 PMC5650056

[hbm24568-bib-0060] Mole, J. P. , Subramanian, L. , Bracht, T. , Morris, H. , Metzler‐Baddeley, C. , & Linden, D. E. (2016). Increased fractional anisotropy in the motor tracts of Parkinson's disease suggests compensatory neuroplasticity or selective neurodegeneration. European Radiology, 26(10), 3327–3335. 10.1007/s00330-015-4178-1 26780637 PMC5021738

[hbm24568-bib-0061] Moll, N. M. , Rietsch, A. M. , Thomas, S. , Ransohoff, A. J. , Lee, J. C. , Fox, R. , … Fisher, E. (2011). Multiple sclerosis normal‐appearing white matter: Pathology‐imaging correlations. Annals of Neurology, 70(5), 764–773. 10.1002/ana.22521 22162059 PMC3241216

[hbm24568-bib-0062] Mottershead, J. P. , Schmierer, K. , Clemence, M. , Thornton, J. S. , Scaravilli, F. , Barker, G. J. , … Miller, D. H. (2003). High field MRI correlates of myelin content and axonal density in multiple sclerosis. A post‐mortem study of the spinal cord. Journal of Neurology, 250, 1293–1301. 10.1007/s00415-003-0192-3 14648144

[hbm24568-bib-0063] Pierpaoli, C. , Chiro, D. , Basser, J. , & Trace, D. (1996). Diffusion tensor MR imaging of the human brain. Radiology, 201, 637–648. 8939209 10.1148/radiology.201.3.8939209

[hbm24568-bib-0064] Polman, C. H. , Reingold, S. C. , Banwell, B. , Clanet, M. , Cohen, J. A. , Filippi, M. , … Connor, P. O. (2011). Diagnostic criteria for multiple sclerosis: 2010 revisions to the McDonald criteria. Annual Neurology, 69, 292–302. 10.1002/ana.22366 PMC3084507 21387374

[hbm24568-bib-0065] Sahraian, M. , Radue, E. , Haller, S. , & Kappos, L. (2010). Black holes in multiple sclerosis: Definition, evolution, and clinical correlations. Acta Neurologica Scandinavica, 122, 1–8. 10.1111/j.1600-0404.2009.01221.x 20003089

[hbm24568-bib-0066] Schmierer, K. , Scaravilli, F. , Altmann, D. R. , Barker, G. J. , & Miller, D. H. (2004). Magnetization transfer ratio and myelin in postmortem multiple sclerosis brain. Annals of Neurology, 56(3), 407–415. 10.1002/ana.20202 15349868

[hbm24568-bib-0067] Schmierer, K. , Wheeler‐Kingshott, C. A. M. , Boulby, P. A. , Scaravilli, F. , Altmann, D. R. , Barker, G. J. , … Miller, D. H. (2007). Diffusion tensor imaging of post mortem multiple sclerosis brain. Human Brain Mapping Journal, 35(2), 467–477. 10.1016/j.neuroimage.2006.12.010 PMC1892244 17258908

[hbm24568-bib-0068] Schmierer, K. , Wheeler‐Kingshott, C. A. M. , Tozer, D. J. , Boulby, P. A. , Parkes, H. G. , Yousry, T. A. , … Miller, D. H. (2008). Quantitative magnetic resonance of postmortem multiple sclerosis brain before and after fixation. Magnetic Resonance in Medicine, 277, 268–277. 10.1002/mrm.21487 PMC2241759 18228601

[hbm24568-bib-0069] Seewann, A. , Vrenken, H. , van der Valk, P. , Blezer, L. , Knol, D. , Castelijns, J. , … Geurts, J. (2009). Diffusely abnormal white matter in chronic multiple sclerosis. Archives of Neurology, 66(5), 601–609. 19433660 10.1001/archneurol.2009.57

[hbm24568-bib-0070] Shrout, E. , & Fleiss, J. (1979). Intraclass correlations: Uses in assessing rater reliability. Psychological Bulletin, 86(2), 420–428. 18839484 10.1037//0033-2909.86.2.420

[hbm24568-bib-0071] Simons, M. , Misgeld, T. , & Kerschensteiner, M. (2014). A unified cell biological perspective on axon‐myelin injury. Journal of Cell Biology, 206(3), 335–345. 10.1083/jcb.201404154 25092654 PMC4121977

[hbm24568-bib-0072] Smith, S. M. (2002). Fast robust automated brain extraction. Human Brain Mapping, 17, 143–155. 10.1002/hbm.10062 12391568 PMC6871816

[hbm24568-bib-0073] Smith, S. M. , Zhang, Y. , Jenkinson, M. , Chen, J. , Matthews, P. M. , Federico, A. , & Stefano, N. D. (2002). Accurate, robust, and automated longitudinal and cross‐cectional brain change analysis. NeuroImage, 489, 479–489. 10.1006/nimg.2002.1040 12482100

[hbm24568-bib-0074] Song, S.‐K. , Sun, S.‐W. , Ju, W.‐K. , Lin, S.‐J. , Cross, A. H. , & Neufeld, A. H. (2003). Diffusion tensor imaging detects and differentiates axon and myelin degeneration in mouse optic nerve after retinal ischemia. NeuroImage, 20, 1714–1722. 10.1016/j.neuroimage.2003.07.005 14642481

[hbm24568-bib-0075] Song, S.‐K. , Sun, S.‐W. , Ramsbottom, M. J. , Chang, C. , Russell, J. , & Cross, A. H. (2002). Dysmyelination revealed through MRI as increased radial (but unchanged axial) diffusion of water. NeuroImage, 17(3), 1429–1436. 10.1006/nimg.2002.1267 12414282

[hbm24568-bib-0076] Tomassini, V. , Matthews, P. M. , Thompson, A. J. , Fuglø, D. , Geurts, J. J. , Johansen‐Berg, H. , … Barkhof, F. (2012). Neuroplasticity and functional recovery in multiple sclerosis. Nature Reviews Neurology, 8(11), 635–646. 10.1038/nrneurol.2012.179 22986429 PMC3770511

[hbm24568-bib-0077] Tomassini, V. , & Palace, J. (2009). Multiple sclerosis lesions: Insights from imaging techniques. Expert Review Neurotheraphy, 9(9), 1341–1359. 10.1586/ERN.09.83 19769449

[hbm24568-bib-0078] Trapp, B. D. , Peterson, J. W. , Ransohoff, R. M. , Rudick, R. A. , Mörk, S. , & Bö, L. (1998). Axonal transection in the lesions of multiple sclerosis. The New England Journal of Medicine, 338(5), 278–285. 10.1056/NEJM199801293380502 9445407

[hbm24568-bib-0079] Trapp, B. D. , & Stys, P. K. (2009). Virtual hypoxia and chronic necrosis of demyelinated axons in multiple sclerosis. The Lancet Neurology, 8(3), 280–291. 10.1016/S1474-4422(09)70043-2 19233038

[hbm24568-bib-0080] Underhill, H. R. , Yuan, C. , & Yarnykh, V. L. (2009). Direct quantitative comparison between cross‐relaxation imaging and diffusion tensor imaging of the human brain at 3.0 T. NeuroImage, 47(4), 1568–1578. 10.1016/j.neuroimage.2009.05.075 19500678

[hbm24568-bib-0081] Van Waesberghe, J. H. , Kamphorst, W. , De Groot, C. J. , Van Walderveen, M. A. , Castelijns, J. A. , Ravid, R. , … Barkhof, F. (1999). Axonal loss in multiple sclerosis lesions: Magnetic resonance imaging insights into substrates of disability. Annals of Neurology, 46(5), 747–754. 10553992 10.1002/1531-8249(199911)46:5<747::aid-ana10>3.3.co;2-w

[hbm24568-bib-0082] Van Walderveen, M. , Kamphorst, W. , Scheltens, P. , Van Waesberghe, J. , Ravid, R. , Valk, J. , … Barkhof, F. (1998). Histopathologic correlate of hypointense lesions on Tl‐weighted spin‐echo MRI in multiple sclerosis. Neurology, 50(95), 1282–1288. 9595975 10.1212/wnl.50.5.1282

[hbm24568-bib-0083] Vavasour, I. M. , Laule, C. , Li, D. K. B. , Traboulsee, A. L. , & MacKay, A. L. (2011). Is the magnetization transfer ratio a marker for myelin in multiple sclerosis? Journal of Magnetic Resonance Imaging, 33(3), 713–718. 10.1002/jmri.22441 21563257

[hbm24568-bib-0084] Vavasour, I. M. , Li, D. K. B. , Traboulsee, A. L. , Moore, G. R. W. , & Mackay, A. L. (2007). Multi‐parametric MR assessment of T1 black holes in multiple sclerosis: Evidence that myelin loss is not greater in hypointense versus isointense T1 lesions. Journal of Neurology, 254, 1653–1659. 10.1007/s00415-007-0604-x 17934875

[hbm24568-bib-0085] Zhang, H. , Schneider, T. , Wheeler‐Kingshott, C. a. , & Alexander, D. C. (2012). NODDI: Practical in vivo neurite orientation dispersion and density imaging of the human brain. NeuroImage, 61(4), 1000–1016. 10.1016/j.neuroimage.2012.03.072 22484410

[hbm24568-bib-0086] Zhang, Y. , Brady, M. , & Smith, S. (2001). Segmentation of brain MR images through a hidden Markov random field model and the expectation‐maximization algorithm. IEEE Transactions on Medical Imaging, 20(1), 45–57. 11293691 10.1109/42.906424

[hbm24568-bib-0087] Zijdenbos, A. P. , Member, S. , Dawant, B. M. , Margolin, R. A. , & Palmer, A. C. (1994). Morphometric analysis of white matter lesions in MR images: Method and validation. IEEE Transactions on Medical Imaging, 13(4), 716–724. 10.1109/42.363096 18218550

[hbm24568-bib-0088] Zinadinov, R. , & Bakshi, R. (2004). Role of MRI in multiple sclerosis I: Inflammation and lesions. Frontiers in Bioscience, 9, 665–683. 14766399 10.2741/1251

