# Supplementary material for: Comparing MRI metrics to quantify white matter microstructural damage in multiple sclerosis
Source: Hum Brain Mapp. 2019 Mar 19;40(10):2917–32. doi: 10.1002/hbm.24568 (PMC6563497; doi:10.1002/hbm.24568)

5 Supplementary material

Figure 1: **Changes in CSF volume fraction (CSFV).** A boxplot across the included 105 patients is shown for global normalised (median z-score) CSF volume fraction within each tissue-state for each patient. Mean z-scores are 0.15±0.34 for *NAWM*, 1.16 ± 0.77 for *T2L*, and 1.93 ± 4.48 for *T1L*. In *NAWM* CSF volume fraction was significantly different from 0 (t = 4.5, $p < .0001$), while it was significantly higher in *T2L* than in *NAWM* (t = 18.1, $p < .0001$). In *T1L* CSF volume fraction was significantly higher than in *T2L* (t = -13.3, $p < .0001$). **Acronyms:** *NAWM* = normal appearing white matter, *T2L* = T2-hyperintense only lesional tissue, *T1L* = T2-hyperintense lesional tissue that appears also T1-hypointense.

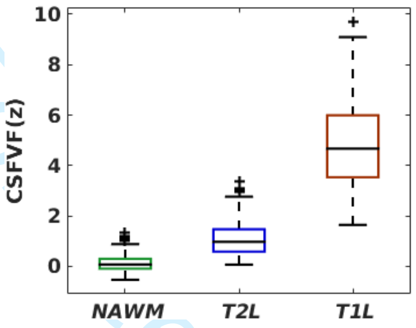

Figure 2: **ROC analysis for DTI-based metrics.** For each patient, a ROC curve was computed for each classification problem: *T2L* vs *NAWM* (left plot), *T1L* vs *NAWM* (middle plot) and *T1L vs T2L* (right plot). The patient-averaged ROC curve (average true positive rate depending on the fixed false positive rate) is plotted for each metric. For the classification problem *T2L* vs *NAWM*, on average RD performed significantly better than AD ($p < .0001$) or FA ($p = .02$). AD performed significantly better than FA ($p = .02$) or RD ($p < .0001$). For the classification problem *T1L* vs *NAWM*, on average RD performed significantly better than FA ($p < .0001$) and AD ($p < .0001$), with no performance differences between FA and AD ($p = .12$). For the classification problem *T1L vs T2L*, on average RD performed significantly better than FA ($p < .0001$) and AD ($p < .0001$). AD performed significantly better than FA ($p = .01$). **Acronyms:** *NAWM* = normal appearing white matter, *T2L* = T2-hyperintense only lesional tissue, *T1L* = T2-hyperintense lesional tissue that appears also T1-hypointense, FA(z) = z-score for fractional anisotropy, RD(z) = z-score for radial diffusivity, MTR(z) = z-score for magnetisation transfer ratio, MWF(z) = z-score for myelin water fraction, AD(z) = z-score for axial diffusivity.

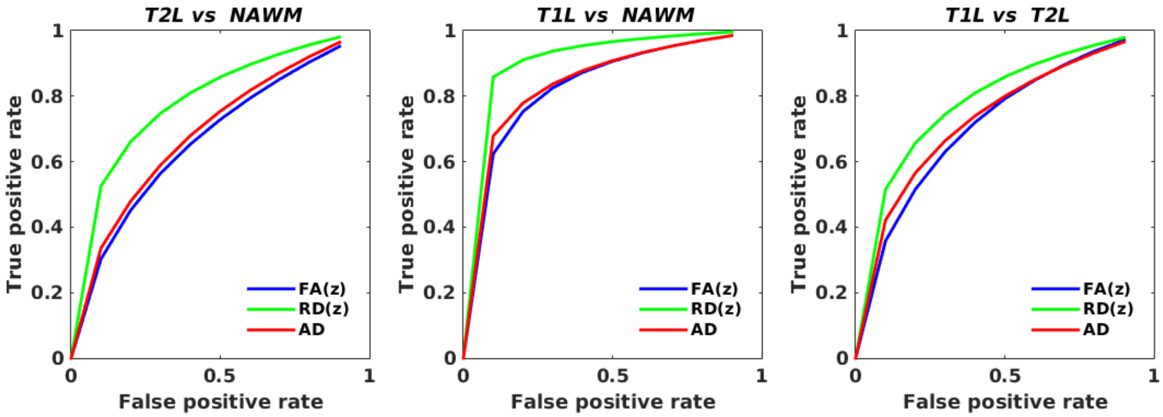

For Peer Review

Figure 3: **Group-level evaluation of the effects of atrophy and registration algorithm on the normalisation to MNI space. A)** Based on the distribution of normalised grey matter volume (SIENAX, Smith et al., 2002), we split our cohort of patients (N = 134; red line) in those with lower vs higher atrophy. The threshold was established based on the 5th percentile (yellow line) of the normalised grey matter volume distribution in our healthy control cohort (N = 27; green line). **B)** We compared three algorithms linear registration (FSL FLIRT), nonlinear registration (FSL FNIRT) and diffeomorphic registration (ANTs SyN). For each algorithm and each group of patients (healthy controls, patients with lower atrophy, and patients with higher atrophy), we normalised the (lesion filled) T1-weighted images to MNI space and averaged the resulting images across the group. The smoothness across the averaged brain provides an indication of registration success, with the worst alignment resulting in the blurriest average image, and the best alignment resulting in the clearest average image. Visual inspection of the output suggests that FLIRT resulted consistently in the blurriest images and ANTs SyN registration provided the clearest average brains. In comparison to the effects of normalisation method, the effect of atrophy on the average image seems negligible. The visual inspection of the images is corroborated by quantification of the FWHM using AFNIs *3dFWHMx* function, showing highest blurriness for FLIRT (HC: 10.8, lower atrophy: 12.6, higher atrophy: 13.2), less blurriness for FNIRT (HC: 10.5, lower atrophy: 10.4, higher atrophy: 10.3) and least blurriness for ANTs (HC: 7.1, lower atrophy: 8.5, higher atrophy: 8.7).

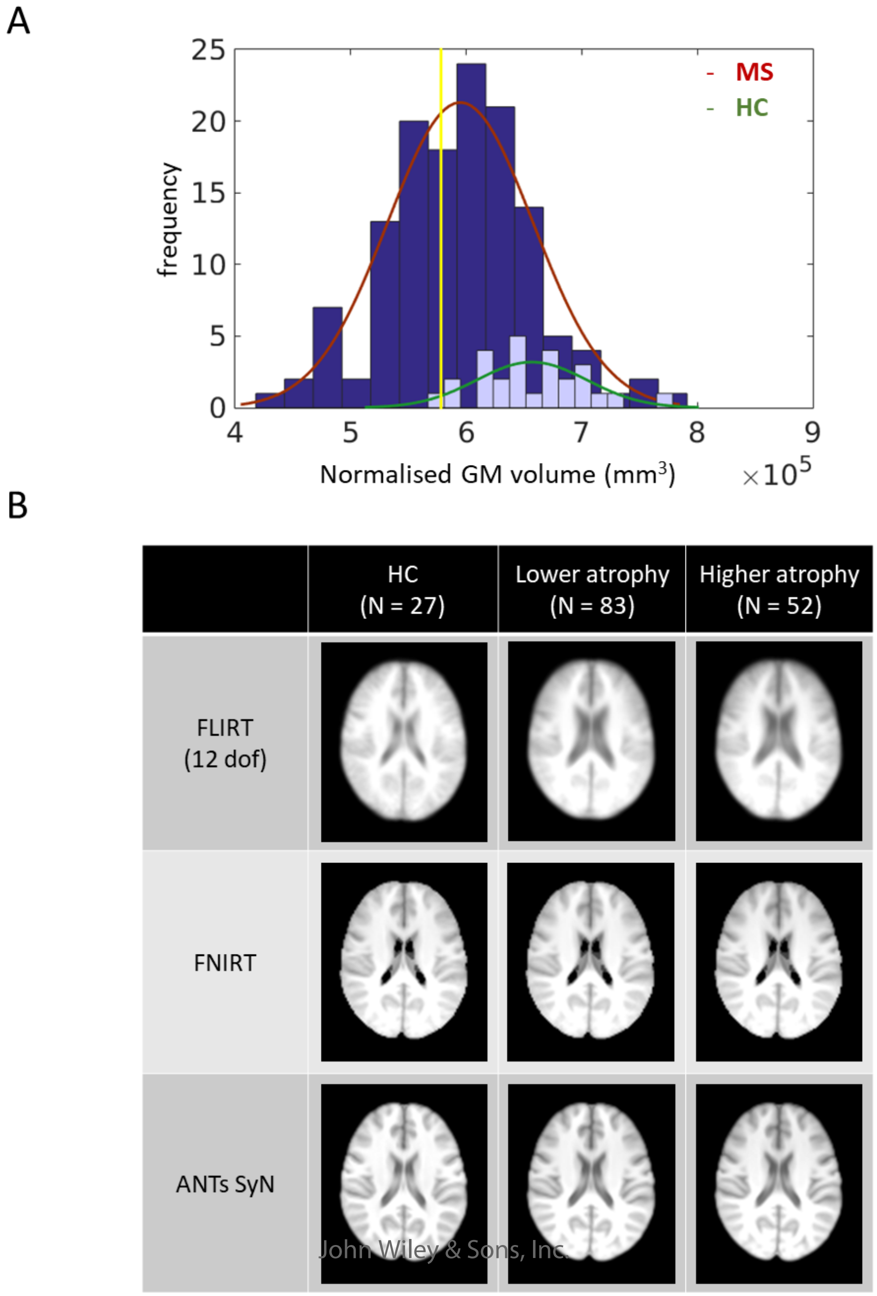

Supplement: Supplementary file 1 — Fig. 1 Changes in CSF volume fraction (CSFV). A boxplot across the included 105 patients is shown for global normalised (median z‐score) CSF volume fraction within each tissue‐state for each patient. Mean z‐scores are 0.15 ± 0.34 for NAWM, 1.16 ± 0.77 for T2L, and 1.93 ± 4.48 for T1L. In NAWM CSF volume fraction was significantly different from 0 (t = 4.5, p < 0.0001), while it was significantly higher in T2L than in NAWM (t = 18.1, p < 0.0001). In T1L CSF volume fraction was significantly higher than in T2L (t = −13.3, p < 0.0001). Acronyms: NAWM = normal appearing white matter, T2L = T2‐hyperintense only lesional tissue, T1L = T2‐hyperintense lesional tissue that appears also T1‐hypointense. Fig. 2 ROC analysis for DTI‐based metrics. For each patient, a ROC curve was computed for each classification problem: T2L vs NAWM (left plot), T1L vs NAWM (middle plot) and T1L vs T2L (right plot). The patient‐averaged ROC curve (average true positive rate depending on the fixed false positive rate) is plotted for each metric. For the classification problem T2L vs NAWM, on average RD performed significantly better than AD (p < 0.0001) or FA (p = 0.02). AD performed significantly better than FA (p = 0.02) or RD (p < 0.0001). For the classification problem T1L vs NAWM, on average RD performed significantly better than FA (p < 0.0001) and AD (p < 0.0001), with no performance differences between FA and AD (p = 0.12). For the classification problem T1L vs T2L, on average RD performed significantly better than FA (p < 0.0001) and AD (p < 0.0001). AD performed significantly better than FA (p = 0.01). Acronyms: NAWM = normal appearing white matter, T2L = T2‐hyperintense only lesional tissue, T1L = T2‐hyperintense lesional tissue that appears also T1‐hypointense, FA(z) = z‐score for fractional anisotropy, RD(z) = z‐score for radial diffusivity, MTR(z) = z‐score for magnetisation transfer ratio, MWF(z) = z‐score for myelin water fraction, AD(z) = z‐score for axial diffusivity. Fig. 3 G [file HBM-40-2917-s001.pdf]
